# Supplementary material for: 4-alkyl-L-(Dehydro)proline biosynthesis in actinobacteria involves N-terminal nucleophile-hydrolase activity of γ-glutamyltranspeptidase homolog for C-C bond cleavage
Source: Nat Commun. 2017 Jul 14;8:16109. doi: 10.1038/ncomms16109 (PMC5519988; doi:10.1038/ncomms16109)
Supplement: Supplementary Information [file ncomms16109-s1.pdf]

SI GUIDE

Type of file: pdf
Size of file: 4,459 KB
Title of file for HTML: Supplementary Information
Description: Supplementary Figures, Supplementary Tables, Supplementary Methods and Supplementary References.

Type of file: pdf
Size of file: 508 KB
Title of file for HTML: Peer Review File
Description:

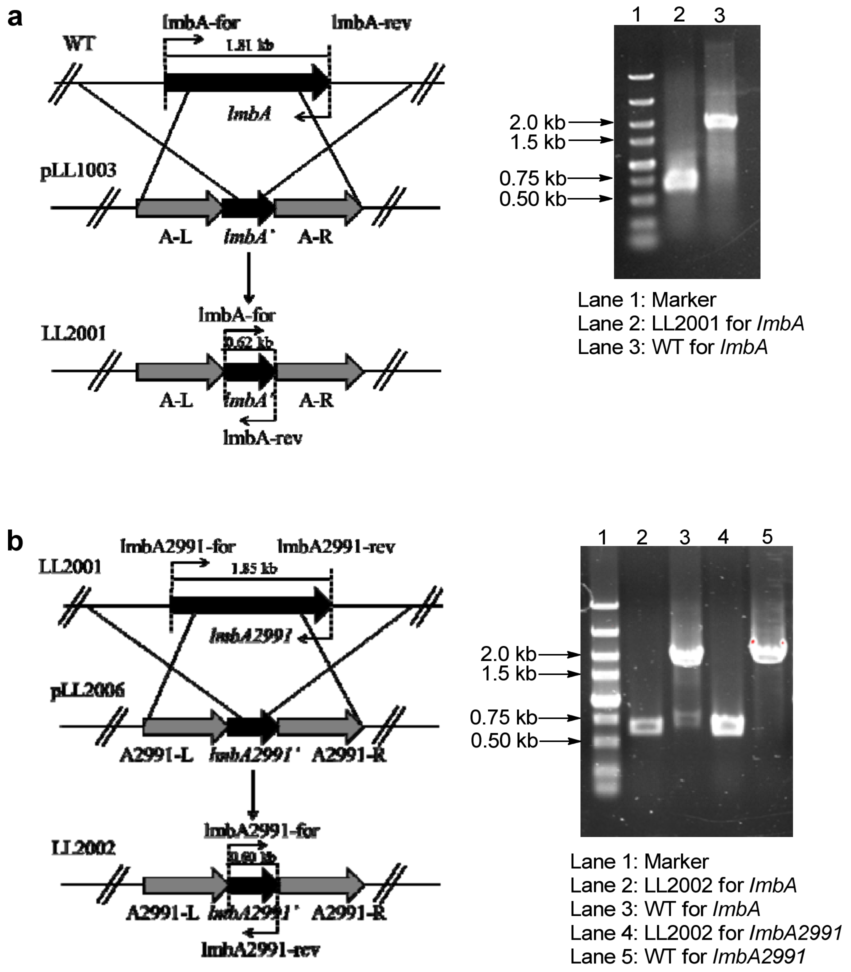

Supplementary Figure 1. Verification of mutant genotypes. (**a**) Construction of the *lmbA* inframe deletion mutant LL2001. The primer pair of lmbA-for and lmbA-rev was used for genotype verification. (**b**) Construction of the *lmbA* and *lmbA2991* double inframe deletions mutant LL2002. The primer pairs of lmbA-for, lmbA-rev and lmbA2991-for, lmbA2991-rev were used for genotype verification.

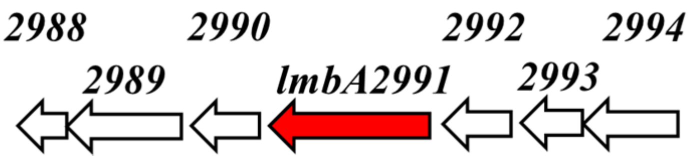

Supplementary Figure 2. Genetic locus of *lmbA2991* (shown in red) in the *S. lincolnensis* ATCC 25466 genome. LmbA2991 is not clustered with the gene cluster coding for lincomycin biosynthesis. The flanking genes *2988*, *2989*, *2990*, *2992*, *2993*, and *2994* share sequence homologies with genes encoding the O-acetyl-ADP-ribose deacetylase (WP_060895150.1) from *S. diastatochromogenes*, the hypothetical protein (WP_053755748.1) from *Streptomyces* sp. MMG1533, the inositol monophosphatase (EHN79732.1) from *S. coelicoflavus* ZG0656, the *S*-adenosyl-methionine (SAM)-dependent methyltransferase (WP_069760310.1) from *Streptomyces* sp. LUP47b, the DNA-binding response regulator (WP_069760309.1) from *Streptomyces* sp. LUP47b and the two-component sensor histidine kinase (WP_030609387.1) from *S. fulvoviolaceus*, respectively.

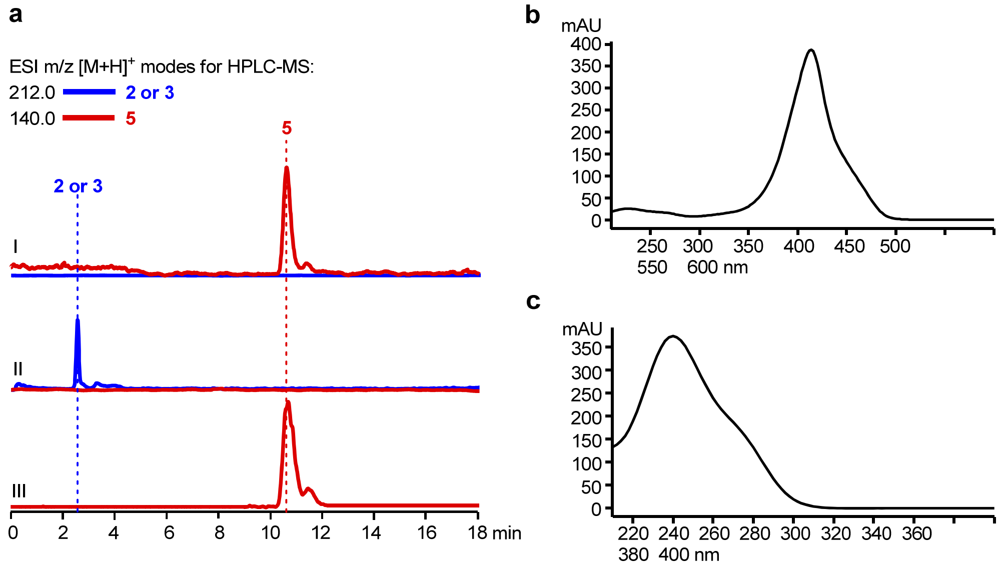

Supplementary Figure 3. Heterologous transformation of L-DOPA.

(**a**) Validation of the catalytic activity of Ant6. I, feeding L-DOPA into *E. coli* BL21(DE3) containing the genes *ant6* and *ant12*; II, feeding L-DOPA into *E. coli* BL21(DE3) containing *ant12* alone; III, **5** standard.

(**b**) Ultraviolet (UV)-visible (Vis) spectrum of **2** or **3**. This spectrum, with a maximal absorption at 414 nm, is highly similar to that reported previously for the mixture of **2** and **3**[1-3]. According to the literatures, neither **2** nor **3** can be purified to homogeneity due to their rapid conversion to each other.

(**c**) UV spectrum of **5**. The maximal absorption is shown at 240 nm, consistent with the synthetic standard.

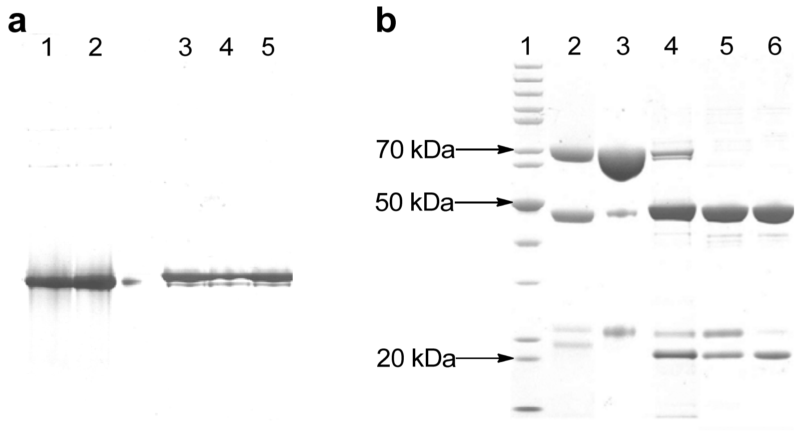

Supplementary Figure 4. Comparative PAGE analysis of the active LmbA-like proteins/variants. **(a)** Non-denaturing gradient PAGE analysis (conc. 4% to 16%). Lane 1, Ant6 wild-type; Lane 2, Ant6-T430S; Lane 3, LmbA2991 wild-type; Lane 4, LmbA2991 HD L&S; Lane 5, LmbA2991 HD L-D420A&S. **(b)** Denaturing SDS-PAGE analysis (conc. 10%). Lane 1, protein standard; Lane 2, Ant6 wild-type; Lane 3, Ant6-T430S; Lane 4, LmbA2991 wild-type; Lane 5, LmbA2991 HD L&S; Lane 6, LmbA2991 HD L-D420A&S.

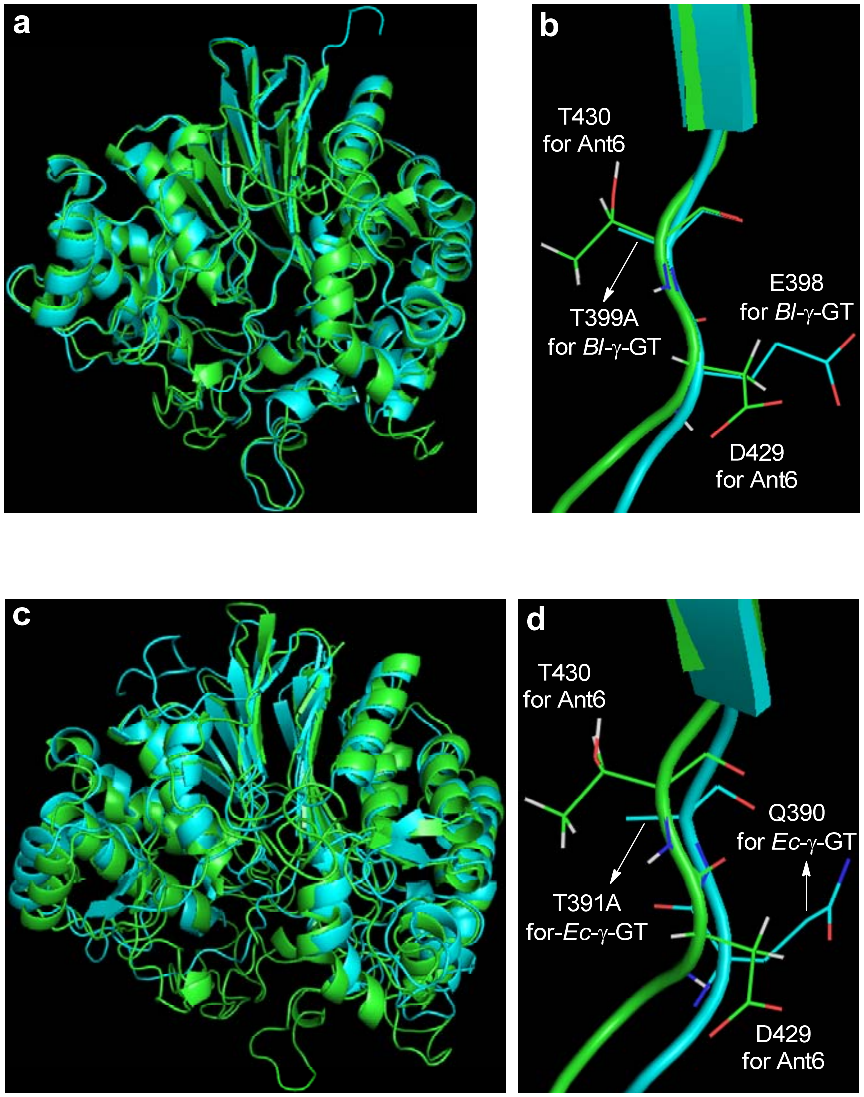

Supplementary Figure 5. Homology modeling of Ant6 performed by I-TASSER.

(**a**) The alignment of Ant6 (Green) and *Bl*-γ-GT T399A (Blue, PDB ID: 4Y23).

(**b**) Partial enlarged detail of Ant6 (Green) and *Bl*-γ-GT T399A (Blue) at the autoproteolytic sites. The related residues (indicated in sticks) of the two proteins are close in space.

(**c**) The alignment of Ant6 (Green) and *Ec*-γ-GT T391A (Blue, PDB ID: 2E0W).

(**d**) Partial enlarged detail of Ant6 (Green) and *Ec*-γ-GT T391A (Blue) at the autoproteolytic sites. The related residues (indicated in sticks) of the two proteins are close in space as well.

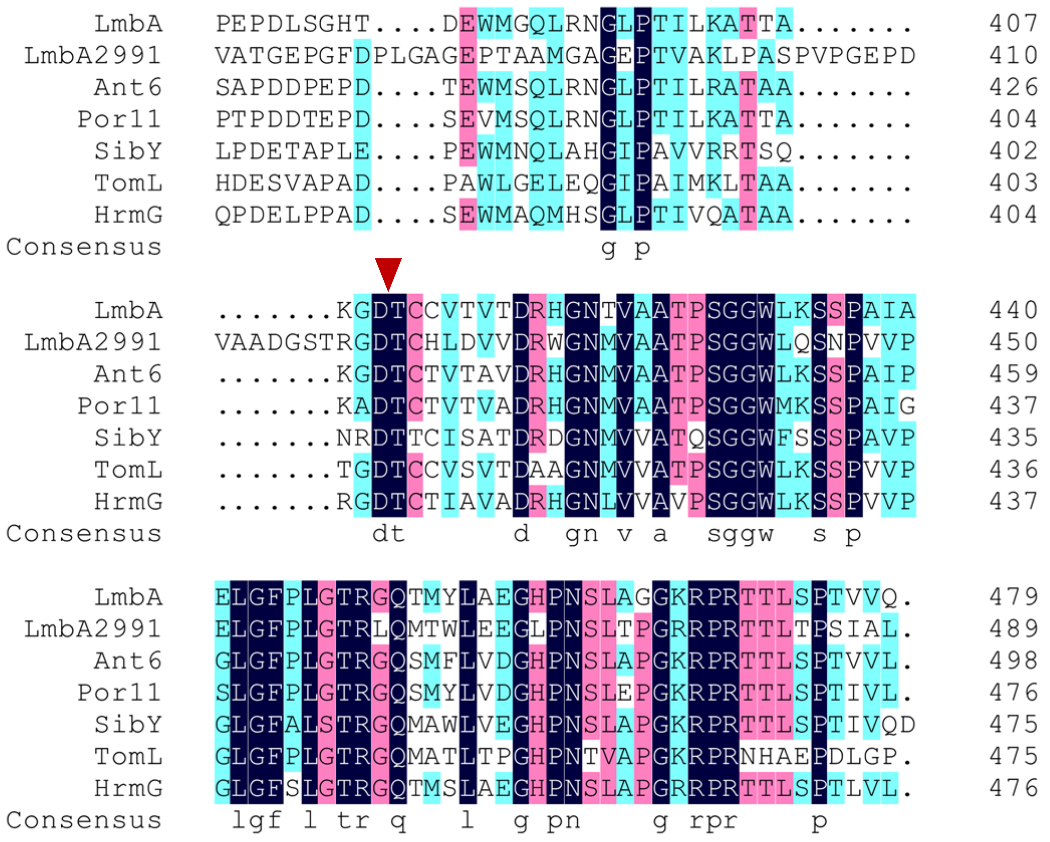

Supplementary Figure 6. Sequence alignment of LmbA and the homologous proteins. Re-examination of the sequence of the gene *hrmG* from *S. griseoflavus* strain W-384 and the flanking regions revealed that its start codon resides at a more upstream region. The corrected open reading frame (ORF) is 1800 bp in length, and 78 bp longer than that originally assigned. The revised sequence of HrmG is used here for alignment. The autoproteolytic sites are indicated by red triangle.

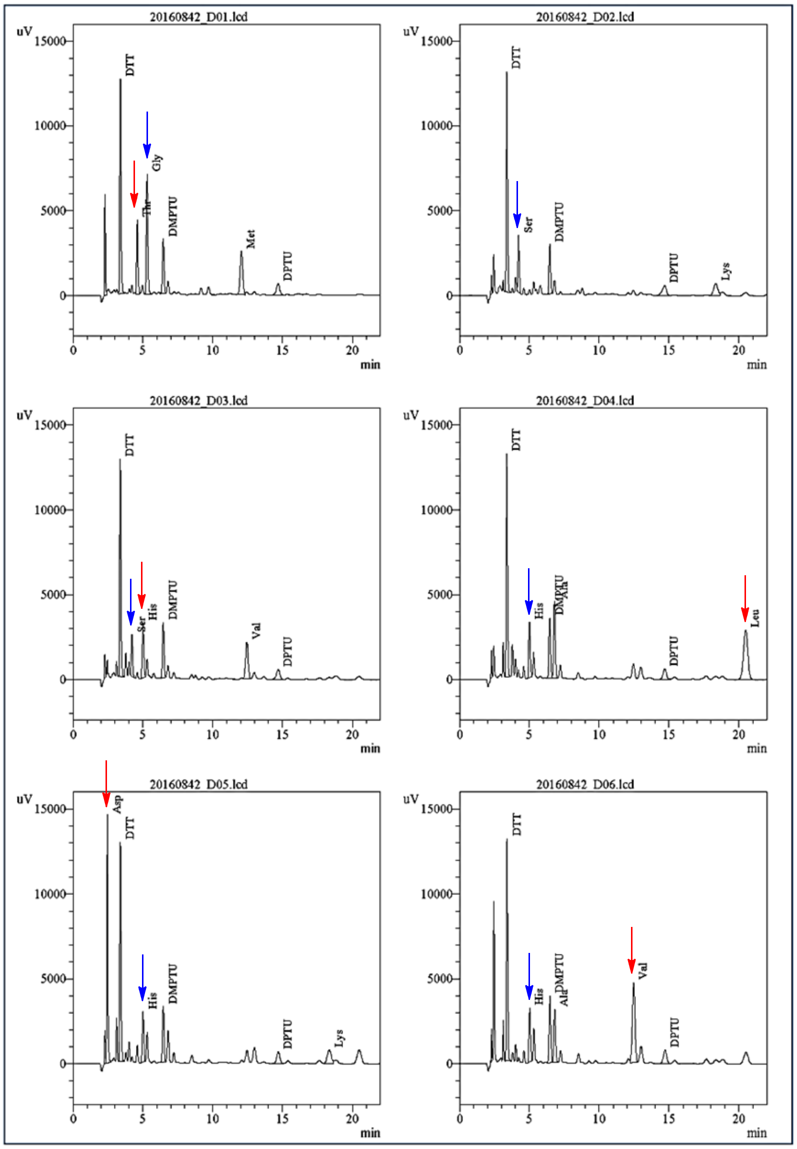
20160842_D01.lcd
uV
15000
10000
5000
0
DTT
Gly
Thr
DMPTU
Met
DPTU
0
5
10
15
20
min
20160842_D02.lcd
DTT
Ser
DMPTU
DPTU
Lys
20160842_D03.lcd
DTT
Ser
His
DMPTU
Val
DPTU
20160842_D04.lcd
DTT
His
DMPTU
Ala
DPTU
Leu
20160842_D05.lcd
Asp
DTT
His
DMPTU
DPTU
Lys
20160842_D06.lcd
DTT
His
Ala
DMPTU
Val
DPTU

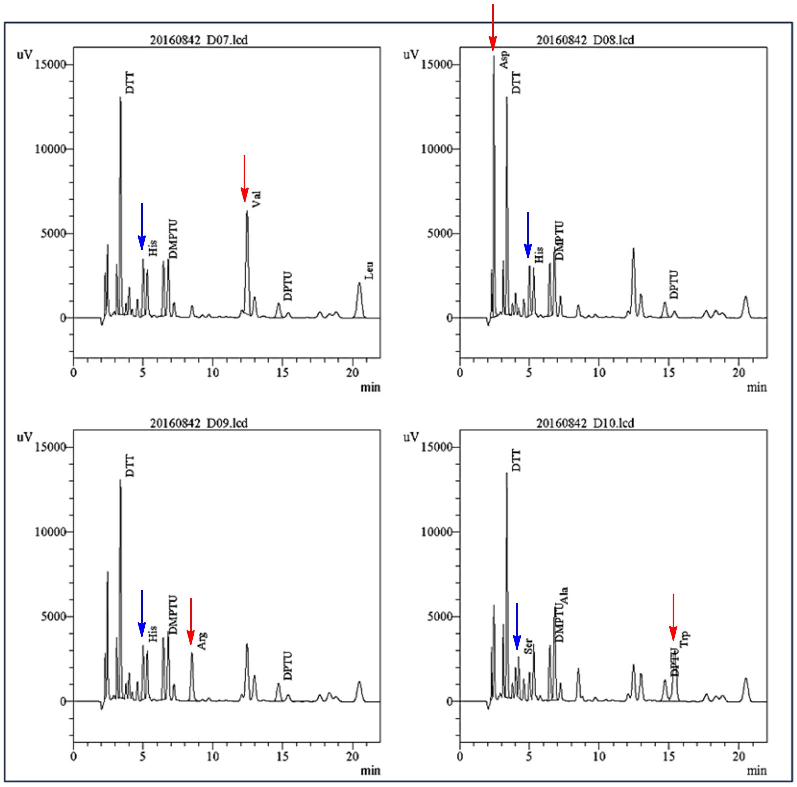

Supplementary Figure 7. *N*-terminal sequencing of LmbA2991 using Edman degradation. The residues shared by the full-length precursor and the large subunit are indicated by blue arrows. The residues for the small subunit are indicated by red arrows. The second residue of the small subunit is Cys, which could not be shown in the result.

The sequence GSSHHHHHHS is for both the full-length precursor and the 420-aa large subunit;

The sequence TCHLDVVDRW is specific for the 197-aa small subunit.

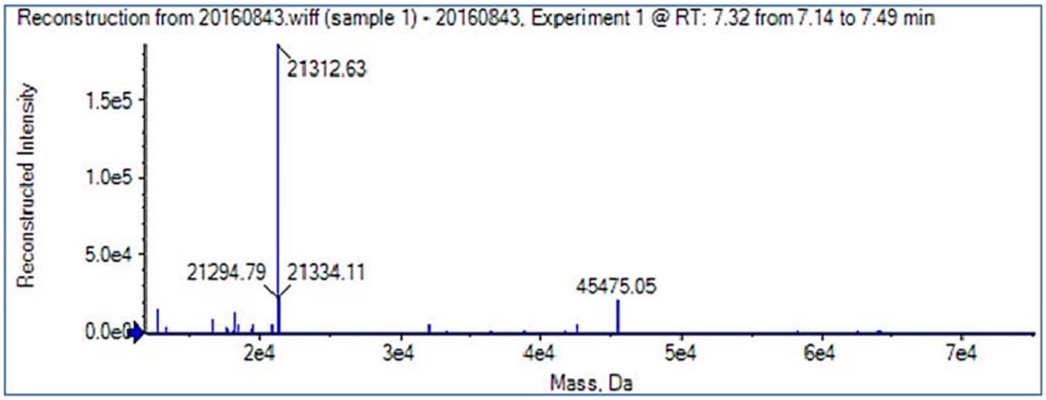

Supplementary Figure 8. HR-MS spectrum of the small subunit in LmbA2991 heterodimer. The exact mass of LmbA2991 small subunit is 21312.16 Da.

a

b

c

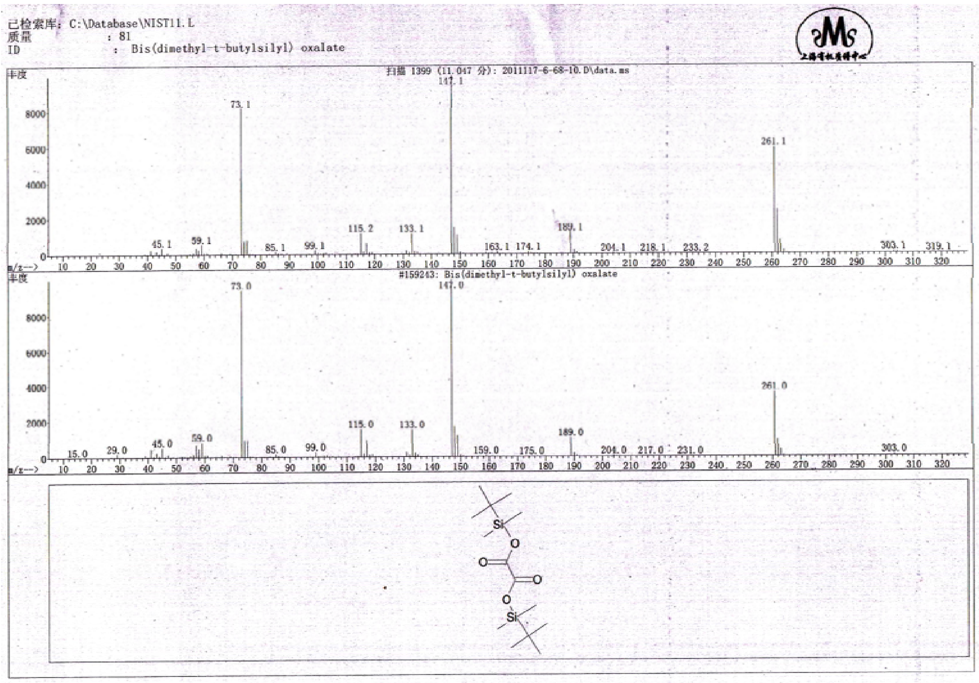

d

National Center for Organic Mass Spectrometry in Shanghai
Shanghai Institute of Organic Chemistry
Chinese Academic of Sciences
High Resolution MS Data Report

Instrument: Waters Micromass GCT Premier   Ionisation Mode: EI+   Electron Energy: 70eV

Card Serial Number: GCT-P-T15-02-0806

Sample Serial Number: 2011117-6-68-10-A

Operator: Li

Date: 2014/02/12

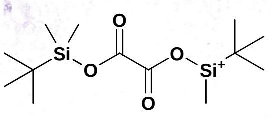

Chemical Formula: $C_{13}H_{27}O_4Si_2^+$
Exact Mass: 303.14424
m/z: 303.14479 (100.0%)

Elemental Composition Report

Single Mass Analysis
Tolerance = 5.0 PPM / DBE: min = -1.5, max = 50.0
Element prediction: Off

Monoisotopic Mass, Odd and Even Electron Ions
627 formula(e) evaluated with 5 results within limits (all results (up to 1000) for each mass)
Elements Used:
C: 0-50 H: 0-80 N: 0-2 O: 0-4 F: 0-3 Si: 0-2

Minimum:				-1.5		
Maximum:		2.0	5.0	50.0		
Mass	Calc. Mass	mDa	PPM	DBE	i-FIT	Formula
303.1449	303.1448	0.1	0.3	2.5	3.0	C13 H27 O4 Si2
	303.1446	0.3	1.0	5.0	1.5	C15 H20 N O2 F3
	303.1443	0.6	2.0	12.0	0.7	C20 H21 N Si
	303.1455	-0.6	-2.0	8.0	0.4	C17 H22 N O F Si
	303.1435	1.4	4.6	9.0	0.7	C18 H19 N O F2

e

National Center for Organic Mass Spectrometry in Shanghai
Shanghai Institute of Organic Chemistry
Chinese Academic of Sciences
High Resolution MS Data Report

Instrument: Waters Micromass GCT Premier   Ionisation Mode: EI+   Electron Energy: 70eV

Card Serial Number: GCT-P-T15-02-0807

Sample Serial Number: 2011117-6-68-10-B

Operator: Li

Date: 2014/02/12

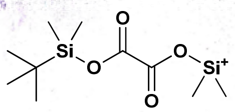

Chemical Formula: $C_{10}H_{21}O_4Si_2^+$
Exact Mass: 261.09729
m/z: 261.09784 (100.0%)

Elemental Composition Report

Single Mass Analysis
Tolerance = 5.0 PPM / DBE: min = -1.5, max = 50.0
Element prediction: Off

Monoisotopic Mass, Odd and Even Electron Ions
537 formula(e) evaluated with 5 results within limits (all results (up to 1000) for each mass)
Elements Used:
C: 0-50 H: 0-80 N: 0-2 O: 0-4 F: 0-3 Si: 0-2

Minimum:				-1.5		
Maximum:		2.0	5.0	50.0		
Mass	Calc. Mass	mDa	PPM	DBE	i-FIT	Formula
261.0976	261.0978	-0.2	-0.8	2.5	8.1	C10 H21 O4 Si2
	261.0974	0.2	0.8	12.0	31.3	C17 H15 N Si
	261.0985	-0.9	-3.4	8.0	46.4	C14 H16 N O F Si
	261.0965	1.1	4.2	9.0	173.1	C15 H13 N O F2
	261.0977	-0.1	-0.4	5.0	225.0	C12 H14 N O2 F3

Supplementary Figure 9. EI-MS and EI-HR-MS spectra of the MTBSTFA derivative **8**. (**a**) EI-MS spectrum of **8**; (**b**) the standard EI-MS spectrum of **8** obtained from the database of National Institute of Standards and Technology (NIST); (**c**) the structure of **8**; (**d**) and (**e**) EI-HR-MS results of **8**.

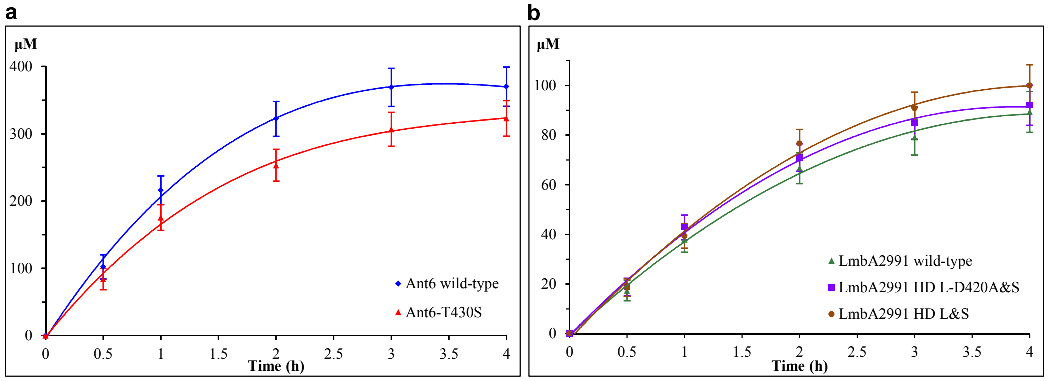

Supplementary Figure 10. Time courses of the formation of **5** for quantitative comparison of Ntn-hydrolase activities of active γ-GT homologs. The large subunit of each γ-GT homolog, which is stable than the small subunit in the heterodimer, served as an indicator to quantify the active form of γ-GT homolog to a same concentration (i.e., 10-µM Ant6 wild-type equal to 30-µM Ant6-T430S and 11.5-µM LmbA2991 wild-type to 10-µM LmbA2991 HD L&S or 10-µM LmbA2991 HD L-D420A&S. The error bars are standard error of mean (n = 3). (**a**) Ant6 wild-type and Ant6-T430S. (**b**) LmbA2991 wild-type, LmbA2991 HD L&S and LmbA2991 HD L-D420A&S.

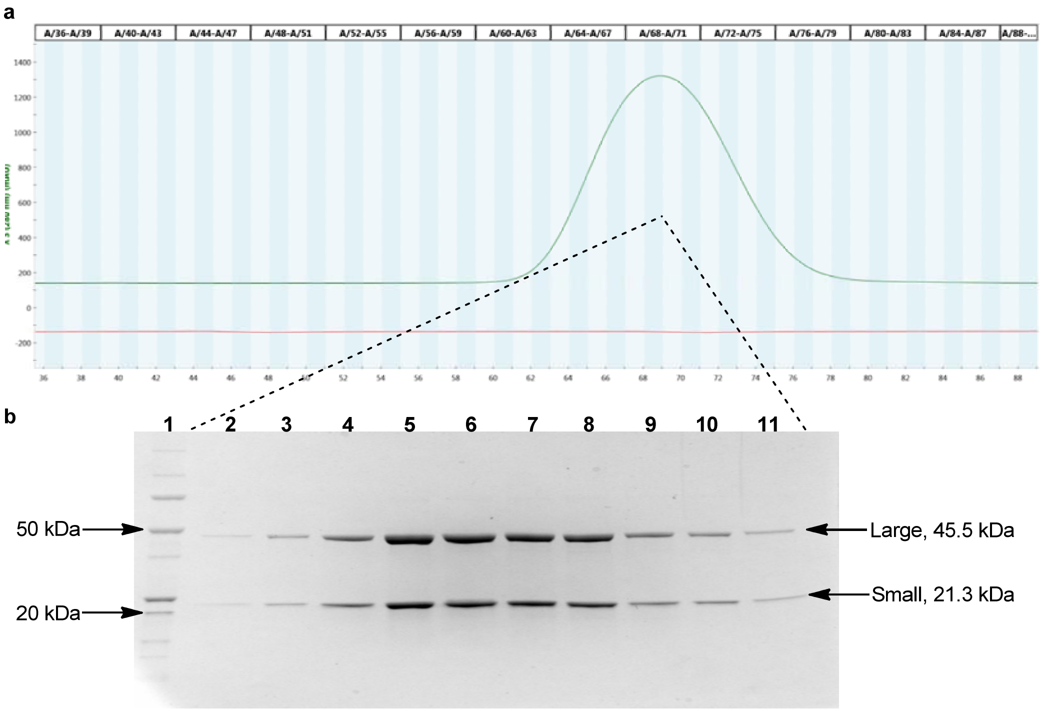

Supplementary Figure 11. Fast protein liquid chromatography (FPLC) analysis of the reconstituted heterodimer LmbA2991 HD L&S. **(a)** FPLC analysis of LmbA2991 HD L&S. **(b)** SDS-PAGE analysis of the LmbA2991 HD L&S elution buffer at different time. Lane 1, protein standard. Lane 2-11, LmbA2991 HD L&S.

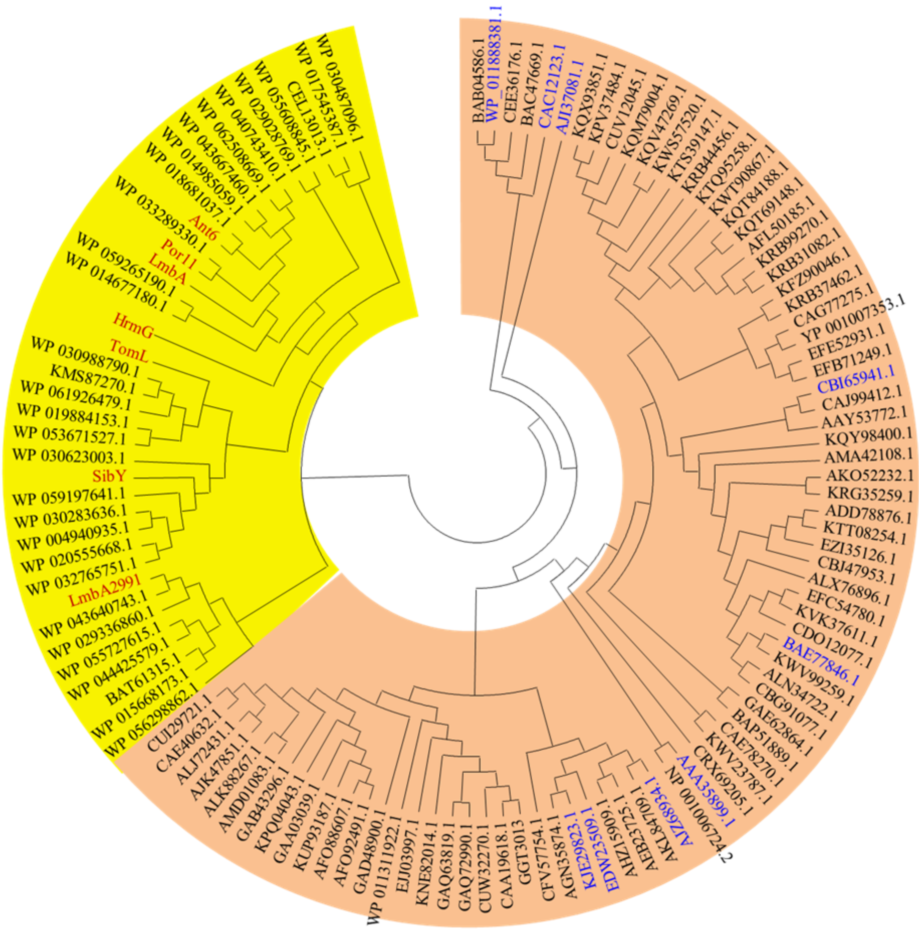

Supplementary Figure 12. Phylogenetic analysis of LmbA-like enzymes and various γ-GT-related proteins. The tree was constructed with the neighbor-joining method. Scale bar, 0.05 substitutions per site. LmbA-like enzymes are indicated by red. γ-GT proteins studied before are indicated by blue.

WP_011888381.1: γ-GT from *Geobacillus thermodenitrificans*; CAC12123.1: γ-GT from *Thermoplasma acidophilum*; AJI37081.1: γ-GT (CapD) from *Bacillus anthracis*; CBI65941.1: γ-GT from *Helicobacter pylori* B8; BAE77846.1: γ-GT from *E. coli* K-12; AAA35899.1: γ-GT from human; AJZ68934.1: γ-GT from *Bacillus subtilis*; EDW23509.1: γ-GT from *Bacillus pumilus* ATCC 7061; KJE29823.1: γ-GT from *Bacillus licheniformis* ATCC 27811.

a

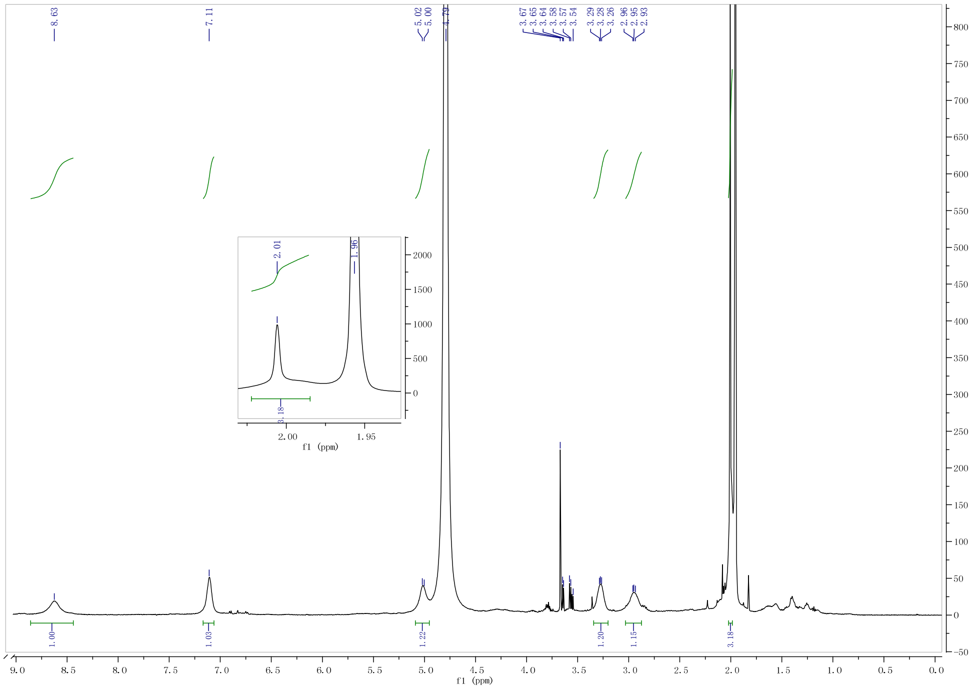

8.63
7.11
5.02
5.00
4.79
3.67
3.65
3.64
3.58
3.57
3.54
3.29
3.28
3.26
2.96
2.95
2.93
2.01
1.96
3.18
2.00
1.95
f1 (ppm)
1.00
1.03
1.22
1.20
1.15
3.18
f1 (ppm)

b

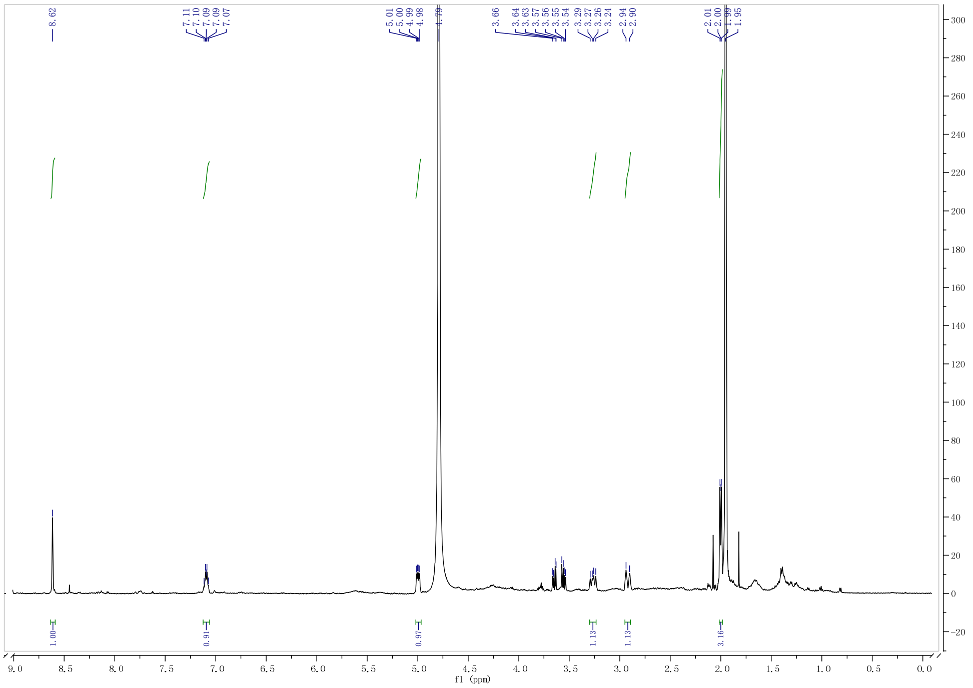

8.62
7.11
7.10
7.09
7.09
7.07
5.01
5.00
4.99
4.98
3.66
3.64
3.63
3.57
3.56
3.55
3.54
3.29
3.27
3.26
3.24
2.94
2.90
2.01
2.00
1.99
1.95
1.00
0.91
0.97
1.13
1.13
3.16
f1 (ppm)

c

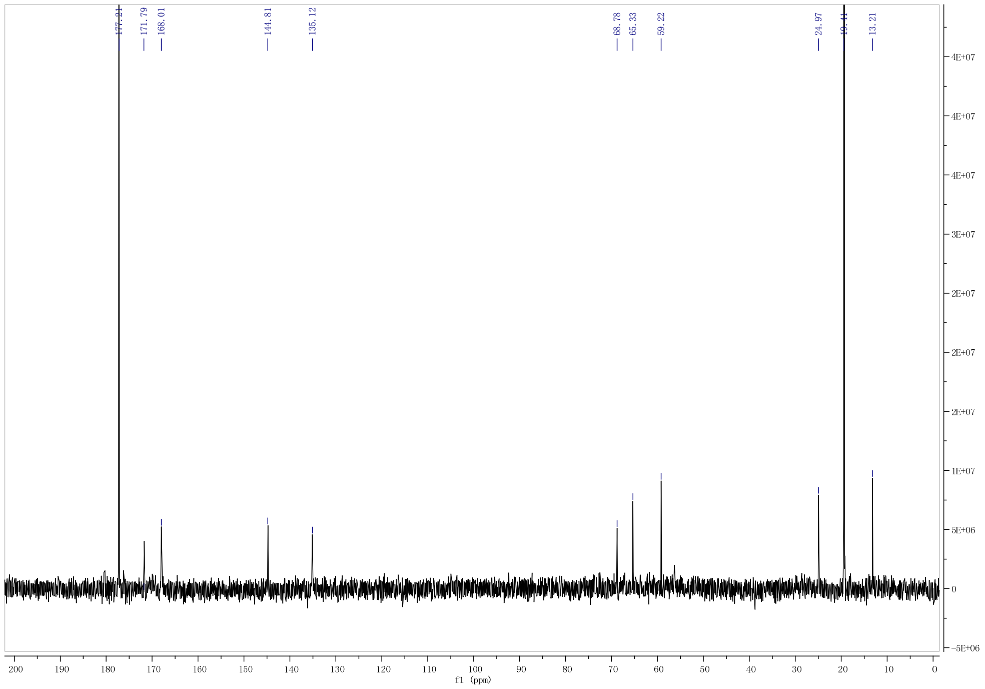

Supplementary Figure 13. NMR spectra of **5**. (**a**) ^{1}H-NMR (500 MHz, D_2O) spectrum of **5** separated from Ant6-catalyzed reaction. (**b**) ^{1}H-NMR (500 MHz, D_2O) spectrum of **5** from the deprotection of **4’**. (**c**) ^{13}C-NMR (125 MHz, D_2O) spectrum of **5** from the deprotection of **4’**. The spectrum of (**a**) is identical to (**b**) with the same chemical shifts and integral areas. δ/ppm=1.95(s) in ^{1}H-NMR and δ/ppm=19.41, 177.21 in ^{13}C-NMR are signals for NH_4OAc which could not be removed completely.

a: ^{1}H-NMR spectrum (500 MHz, $CDCl_3$).

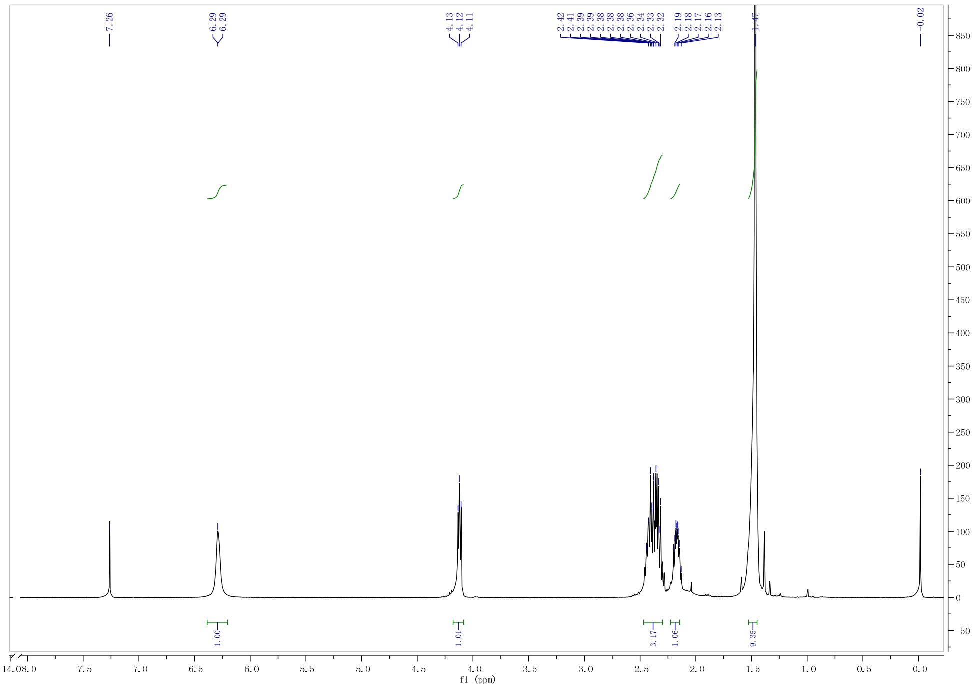

b: ^{13}C-NMR spectrum (125 MHz, $CDCl_3$).

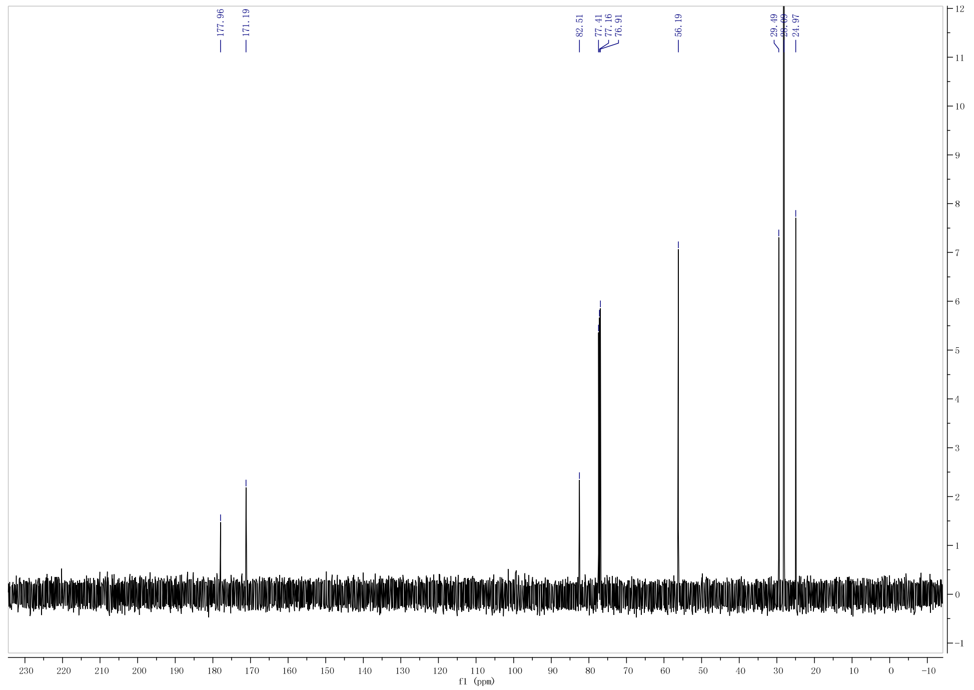

Supplementary Figure 14. NMR spectra of compound **S2**.

a: ^{1}H-NMR spectrum (500 MHz, $CDCl_3$).

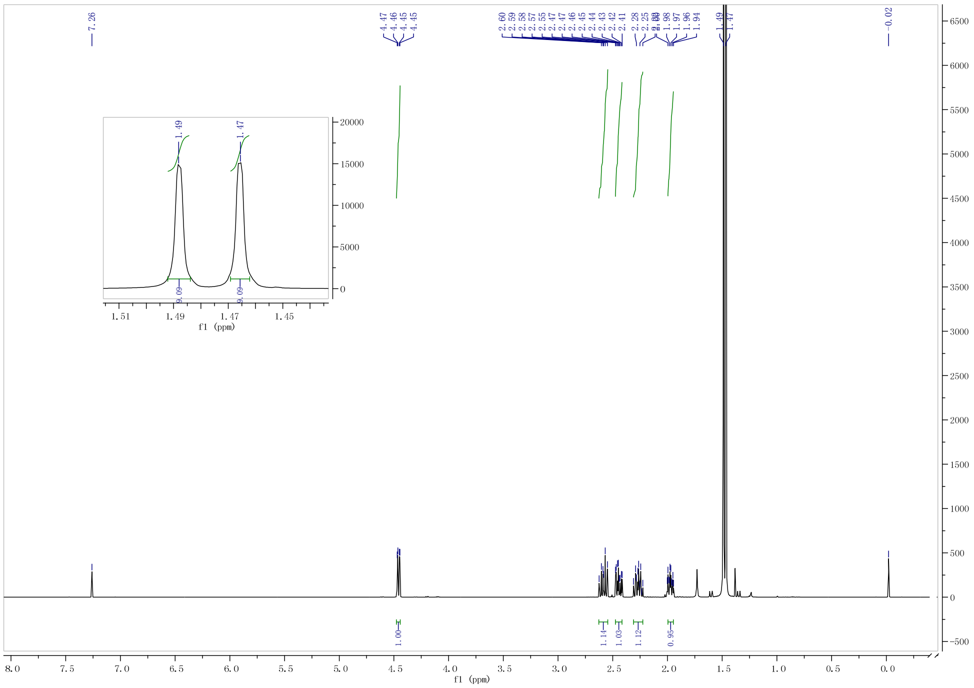

b: ^{13}C-NMR spectrum (125 MHz, $CDCl_3$).

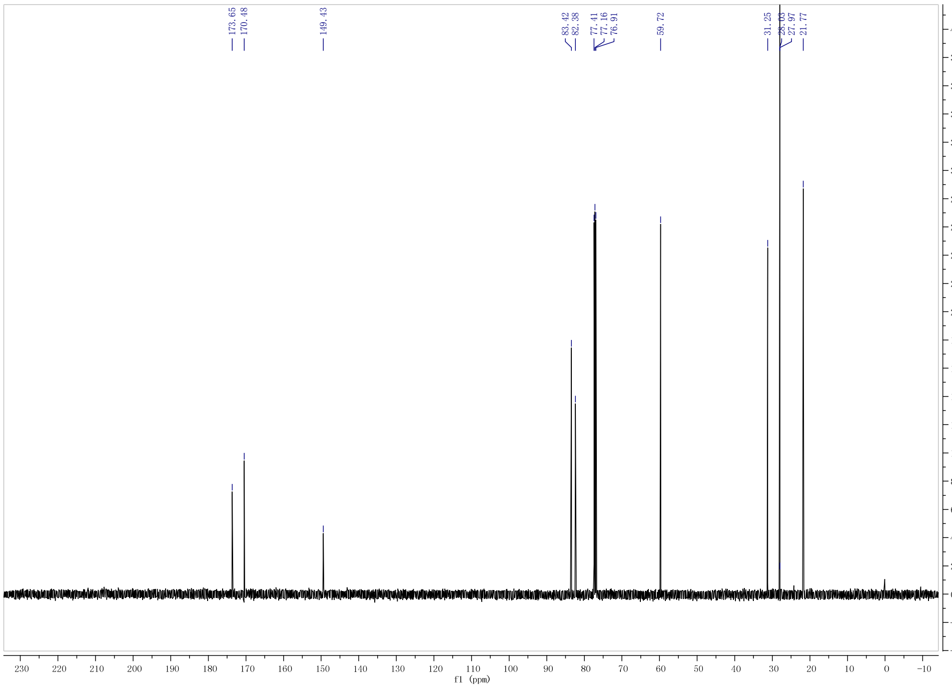

Supplementary Figure 15. NMR spectra of compound **S3**.

a: ^{1}H-NMR spectrum (500 MHz, $(CD_3)_2CO$).

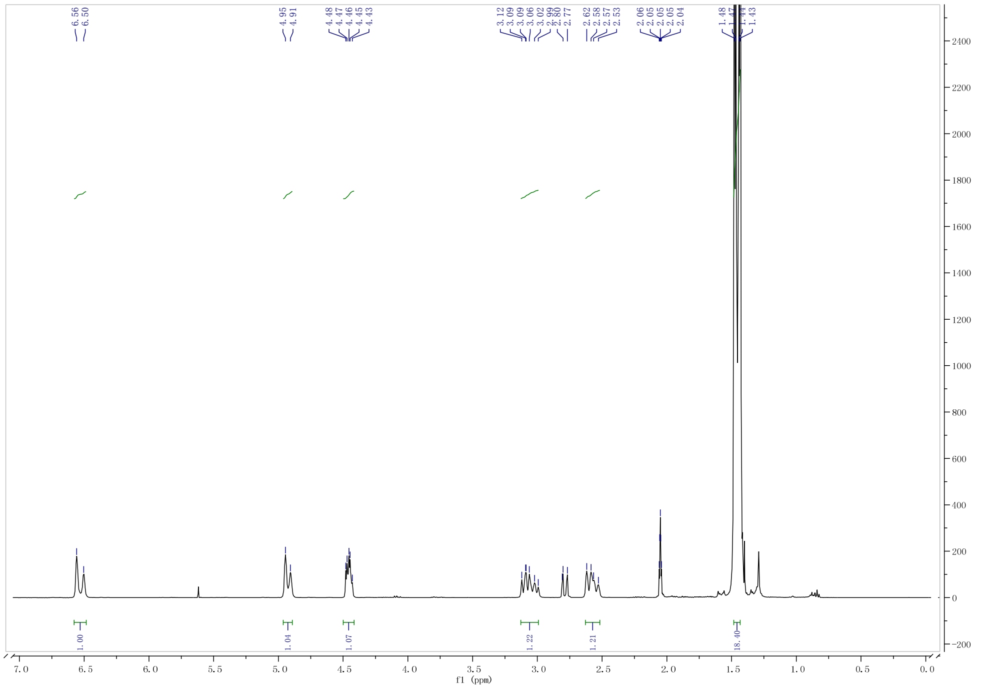

b: ^{13}C-NMR spectrum (125 MHz, $(CD_3)_2CO$).

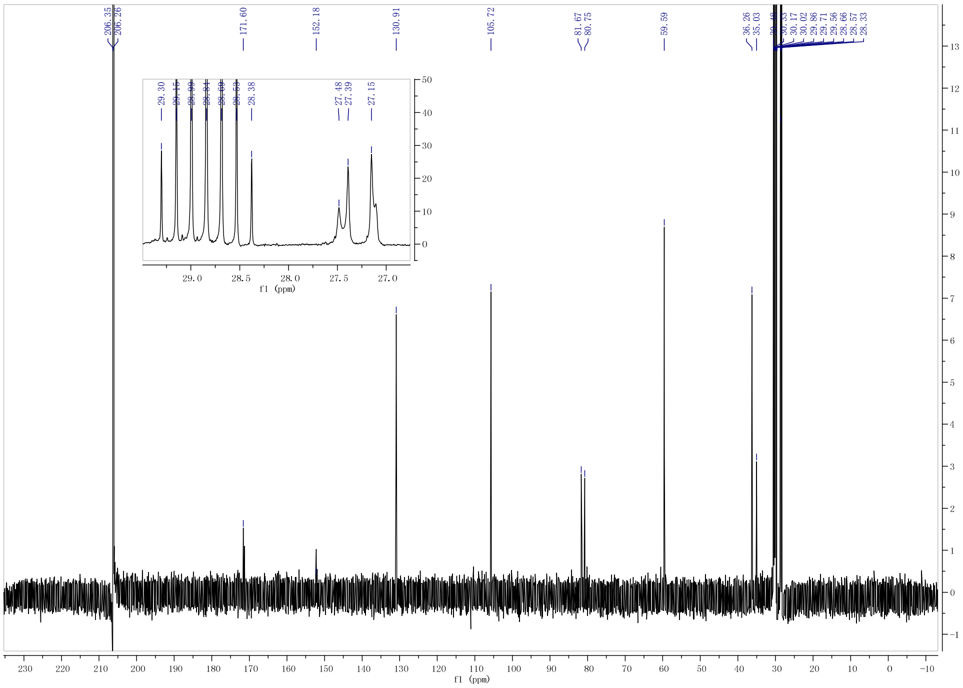

Supplementary Figure 16. NMR spectra of compound **S4**.

a: ^{1}H-NMR spectrum (500 MHz, $CDCl_3$).

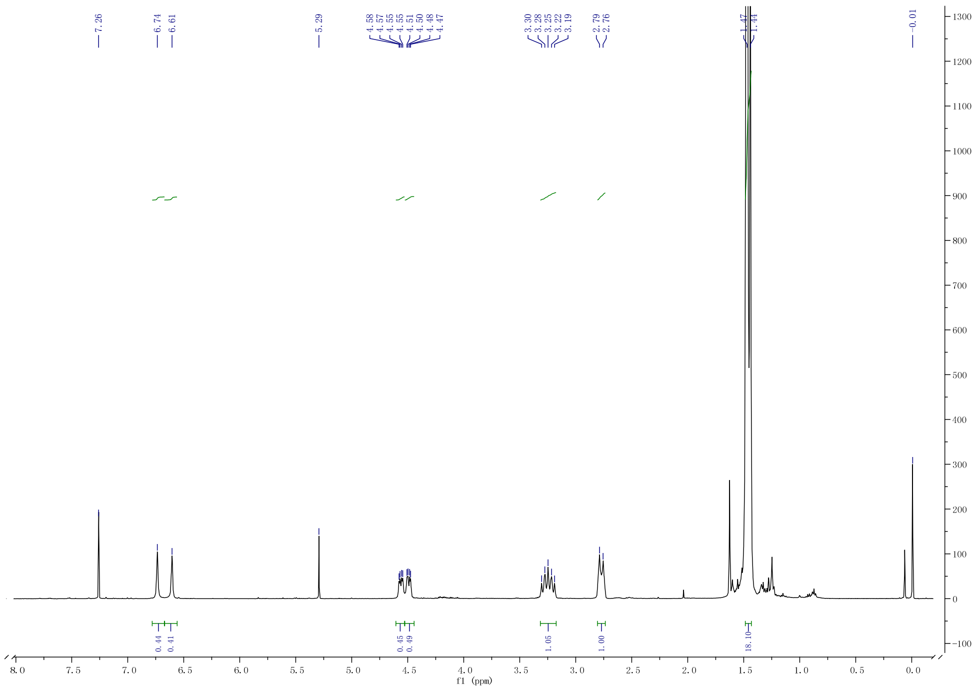

b: ^{13}C-NMR spectrum (125 MHz, $CDCl_3$).

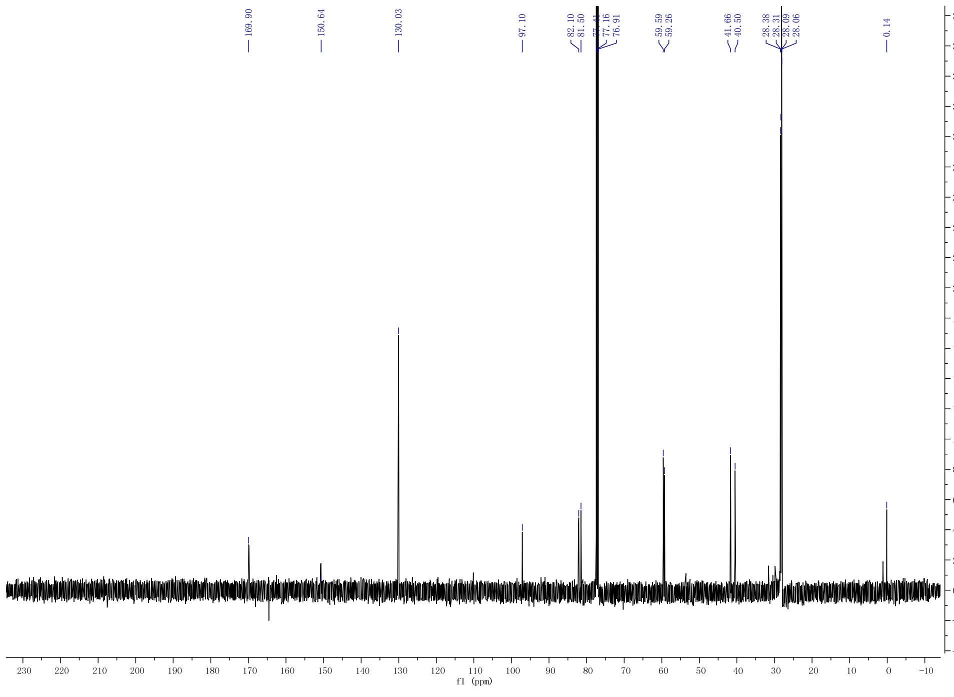

Supplementary Figure 17. NMR spectra of compound **S5**.

a: ^{1}H-NMR spectrum (500 MHz, $CDCl_3$).

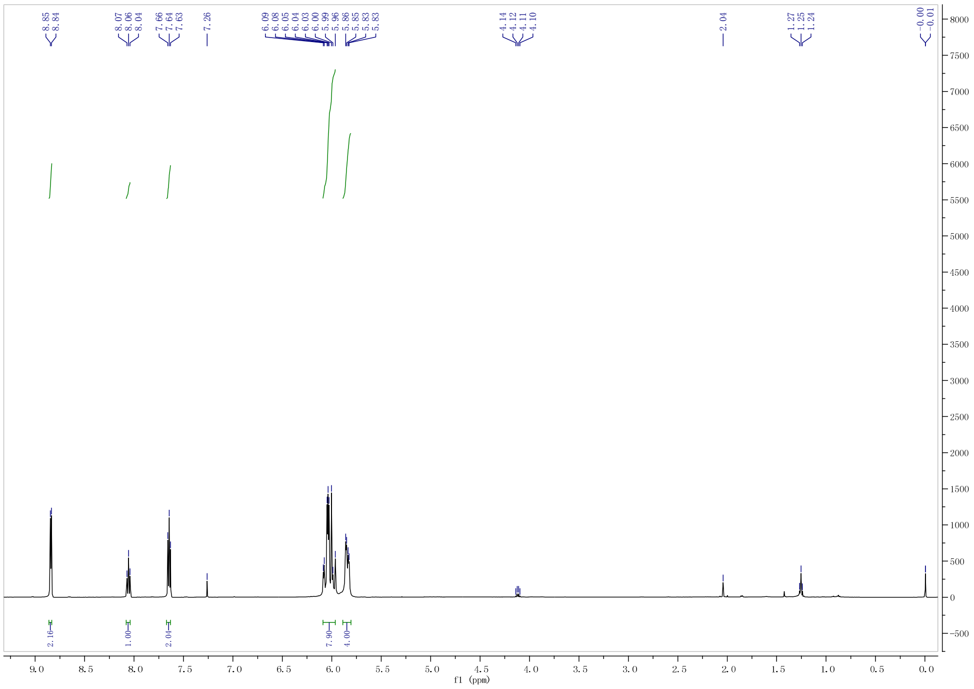

b: ^{13}C-NMR spectrum (125 MHz, $CDCl_3$).

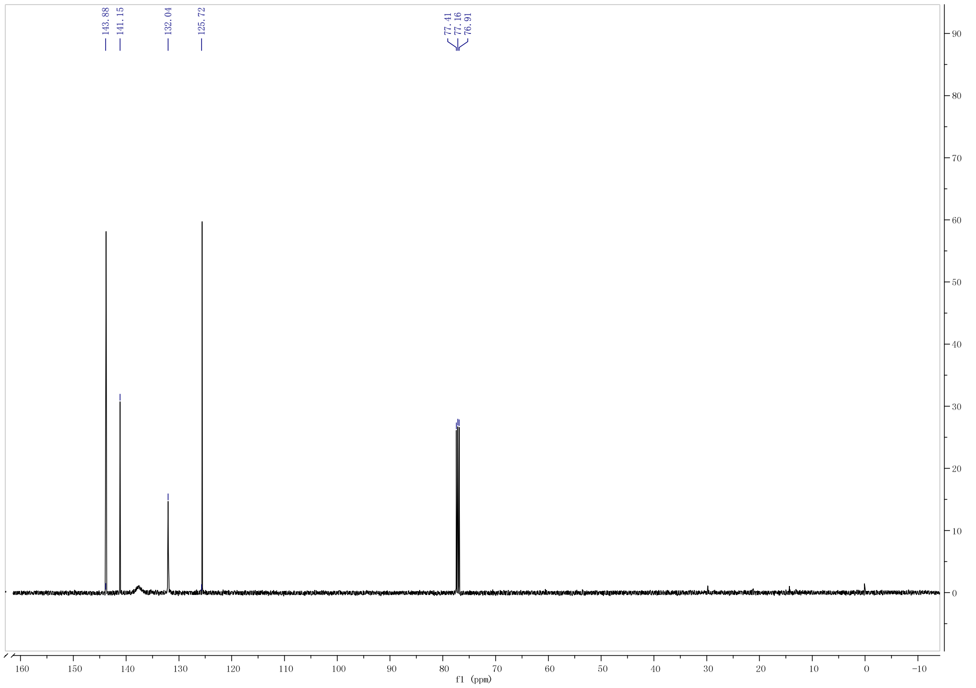

Supplementary Figure 18. NMR spectra of compound **S7**.

a: ^{1}H-NMR spectrum (500 MHz, $CDCl_3$).

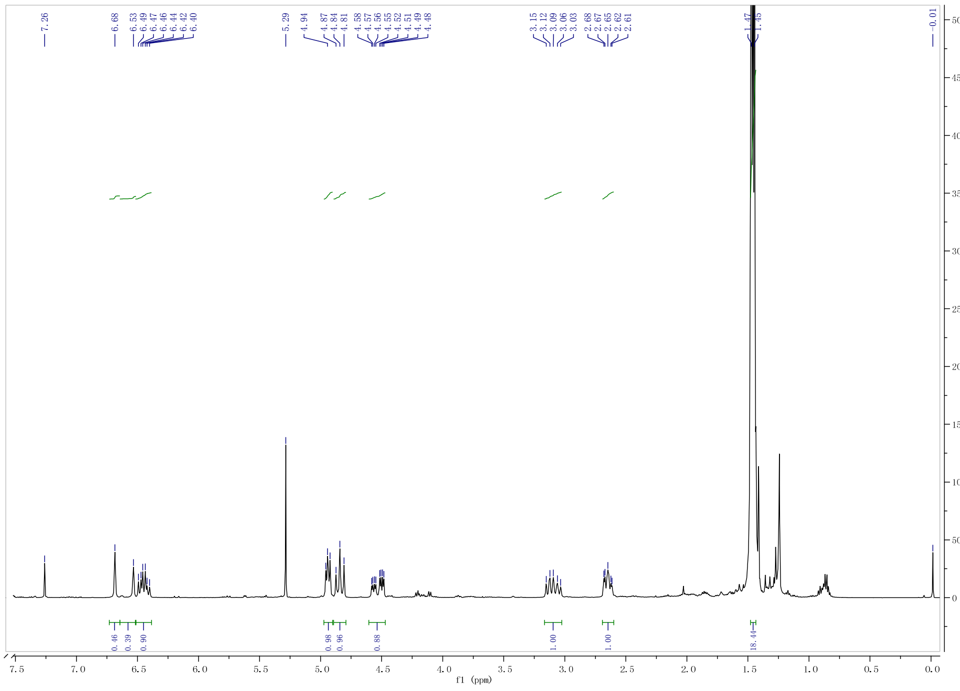

b: ^{13}C-NMR spectrum (125 MHz, $CDCl_3$).

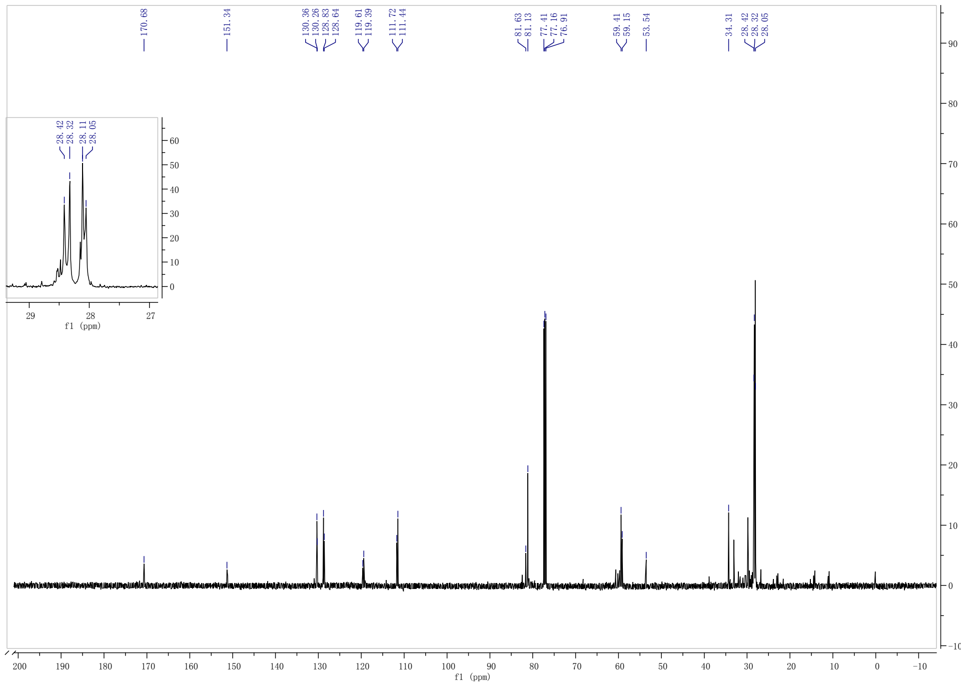

Supplementary Figure 19. NMR spectra of compound **4’**.

Supplementary Table 1. Bacterial strains and plasmids.

Strain/Plasmid	Characteristic(s)	Source/ Reference
S. lincolnensis		
NRRL ISP-5355	Wild type strain, lincomycin-producing, identical to the ATCC 25466 strain	NRRL
LL2001	The *lmbA* in-frame deletion mutant of *S. lincolnensis* NRRL ISP-5355	This study
LL2002	The *lmbA* and *lmbA2991* double in-frame deletion mutant of *S. lincolnensis* NRRL ISP-5355	This study
LL2003	LL2001 derivative carrying *lmbA in trans*	This study
LL2004	LL2001 derivative carrying *ant6 in trans*	This study
S.refuineus		
NRRL 3143	Anthramycin-producing strain	NRRL
Streptosporangi-um Sibircum		
NRRL B-16786	Sibiromycin-producing strain	NRRL
E. coli		
DH5α	Host for general cloning	Invitrogen
ET12567 (pUZ8002)	Donor strain for conjugation between *E.coli* and *Streptomyces*	4
BL21 (DE3)	Host for protein expression	NEB
LL2005	ET12567 (pUZ8002) derivative, containing pLL2003	This study
LL2006	ET12567 (pUZ8002) derivative, containing pLL2006	This study
LL2007	ET12567 (pUZ8002) derivative, containing pLL2008	This study
LL2008	ET12567 (pUZ8002) derivative, containing pLL2010	This study
LL2009	BL21 (ED3) derivative, containing pLL2012 for producing LmbB1	This study
LL2010	BL21 (ED3) derivative, containing pLL2021 for producing Ant6	This study
LL2011	BL21 (ED3) derivative, containing pLL2030 for producing LmbA2991	This study
LL2012	BL21 (ED3) derivative, containing pLL2034 for producing LmbA2991 HD L&S	This study
LL2013	LL2010 derivative, containing pLL2035	This study
LL2014	LL2010 derivative, containing pLL2036	This study
LL2015	LL2010 derivative, containing pLL2037	This study
LL2016	LL2010 derivative, containing pLL2038	This study
LL2017	LL2010 derivative, containing pLL2039	This study
LL2018	LL2010 derivative, containing pLL2040	This study
LL2019	LL2010 derivative, containing pLL2041	This study

LL2020	LL2010 derivative, containing pLL2042	This study
LL2021	LL2010 derivative, containing pLL2043	This study
LL2022	LL2010 derivative, containing pLL2044	This study
LL2023	LL2010 derivative, containing pLL2045	This study
LL2024	LL2010 derivative, containing pLL2046	This study
LL2025	LL2010 derivative, containing pLL2047	This study
LL2026	LL2010 derivative, containing pLL2048	This study
**Plasmids**		
pMD19-T	*E. coli* subcloning vector	Takara
pKC1139	*E.coli-Streptomyces* shuttle vector for gene inactivation and complementary, temperature sensitive replication in *Streptomyces*	5
pET-28a(+)	Protein expression vector used in *E. coli*, encoding *N*-terminal 6× His-tag, kanamycin resistance	Novagen
pACYCDuet-1	Protein co-expression vector used in *E. coli*, encoding *N*-terminal 6× His tag on the former part, chloramphenicol resistance	Novagen
pWHM79	pGEM-3zf derivative carrying a 0.45 kb fragment containing the *PermE** promoter	6
pLL2001	pMD19-T derivative containing partial *lmbA* fragment	This study
pLL2002	pMD19-T derivative containing partial *lmbA* fragment	This study
pLL2003	pKC1139 derivative for *lmbA* in-frame deletion	This study
pLL2004	pMD19-T derivative containing partial *lmbA2991* fragment	This study
pLL2005	pMD19-T derivative containing partial *lmbA2991* fragment	This study
pLL2006	pKC1139 derivative for *lmbA2991* in-frame deletion	This study
pLL2007	pMD19-T derivative containing *lmbA* complemental fragment	This study
pLL2008	pKC1139 derivative for *lmbA* complementation in trans	This study
pLL2009	pMD19-T derivative containing *ant6* complemental fragment	This study
pLL2010	pKC1139 derivative for *ant6* complementation *in trans*	This study
pLL2011	pMD-19T derivative containing *lmbB1* for pLL2012 construction	This study
pLL2012	pET28a(+) derivative containing *lmbB1*	This study
pLL2013	pMD-19T derivative containing *lmbB1* for pLL2016, pLL2017 and pLL2030 construction	This study
pLL2014	pMD-19T derivative containing *lmbA*	This study
pLL2015	pACYCDuet-1 derivative containing *lmbA*	This study
pLL2016	pACYCDuet-1 derivative containing *lmbA* and *lmbB1*	This study
pLL2017	pACYCDuet-1 derivative containing *lmbB1*	This study
pLL2018	pMD-19T derivative containing *ant12*	This study
pLL2019	pMD-19T derivative containing *ant6*	This study
pLL2020	pACYCDuet-1 derivative containing *ant6*	This study
pLL2021	pACYCDuet-1 derivative containing *ant6* and *ant12*	This study
pLL2022	pACYCDuet-1 derivative containing *ant12*	This study

pLL2023	pMD-19T derivative containing *sibV*	This study
pLL2024	pMD-19T derivative containing *sibY*	This study
pLL2025	pACYCDuet-1 derivative containing *sibY*	This study
pLL2026	pACYCDuet-1 derivative containing *sibY* and *sibV*	This study
pLL2027	pACYCDuet-1 derivative containing *sibV*	This study
pLL2028	pMD-19T derivative containing *lmbA2991*	This study
pLL2029	pACYCDuet-1 derivative containing *lmbA2991*	This study
pLL2030	pACYCDuet-1 derivative containing *lmbA2991* and *lmbB1*	This study
pLL2031	pMD-19T derivative containing *lmbA2991* large subunit	This study
pLL2032	pMD-19T derivative containing *lmbA2991* small subunit	This study
pLL2033	pACYCDuet-1 derivative containing *lmbA2991* large subunit	This study
pLL2034	pACYCDuet-1 derivative containing *lmbA2991* HD L&S	This study
pLL2035	pACYCDuet-1 derivative containing *ant6* D429A and *ant12*	This study
pLL2036	pACYCDuet-1 derivative containing *ant6* T430A and *ant12*	This study
pLL2037	pACYCDuet-1 derivative containing *ant6* T430C and *ant12*	This study
pLL2038	pACYCDuet-1 derivative containing *ant6* T430S and *ant12*	This study
pLL2039	pACYCDuet-1 derivative containing *ant6* D429A&T430A and *ant12*	This study
pLL2040	pACYCDuet-1 derivative containing *lmbA2991* D420A and *lmbB1*	This study
pLL2041	pACYCDuet-1 derivative containing *lmbA2991* T421A and *lmbB1*	This study
pLL2042	pACYCDuet-1 derivative containing *lmbA2991* T421C and *lmbB1*	This study
pLL2043	pACYCDuet-1 derivative containing *lmbA2991* T421S and *lmbB1*	This study
pLL2044	pACYCDuet-1 derivative containing *lmbA2991* D420A&T421A and *lmbB1*	This study
pLL2045	pACYCDuet-1 derivative containing *lmbA2991* HD L-420A&S	This study
pLL2046	pACYCDuet-1 derivative containing *lmbA2991* HD L&S-T1A	This study
pLL2047	pACYCDuet-1 derivative containing *lmbA2991* HD L&S-T1C	This study
pLL2048	pACYCDuet-1 derivative containing *lmbA2991* HD L&S-T1S	This study

Supplementary Table 2. Primers used in this study.

Primer	Sequence (5'-3')
lmbA-L-for	TATGAATTCGTAGTGAACATCGCCCTGCCTG EcoRI
lmbA-L-rev	TATTCTAGAGTTGAAGTGGGGTTCCACGAC XbaI
lmbA-R-for	TATTCTAGACAGACGATGTACCTGGCCGAG XbaI
lmbA-R-rev	TATAAGCTTCGTTGTTCCTGGTGCTGGTTC HindIII
lmbA2991-L-for	TATGAATTCGCGGGCGTGCGCGGCGAGCGCGA EcoRI
lmbA2991-L-rev	TATTCTAGACTGCGCGCCCGGGTCCGCGGCGG XbaI
lmbA2991-R-for	TATTCTAGAGCCGGGGACGAGGTCCAGCCCCA XbaI
lmbA2991-R-rev	TATAAGCTTCGGCGGCACCCTGCACACGGGCC HindIII
lmbA-com-for	TATGGATCCCCGACTCCCGGATAACTTCCAG BamHI
lmbA-com-rev	TATTCTAGACGGCGGTCTGGACACCGACATG XbaI
ant6-com-for	TATAGATCTTGCGGTGCTCTACCCGATCGGC BglII
ant6-com-rev	TATTCTAGAGGTCTCGGCGATCGCCCGCACG XbaI
lmbB1-28a-for	TATCATATGCCGTCAGTAAAGTCAATGC NdeI
lmbB1-28a-rev	TATAAGCTTATCGGGGGCCGTCCGGAACGTAG HindIII
lmbA-for	TATGAATTCAATGCTTCCATCGAAGCCGGAGC EcoRI
lmbA-rev	TATAAGCTTTTATCCGCAGATCGCGTAGGGCTGG HindIII
lmbB1-Duet-for	TATCATATGCCGTCAGTAAAGTCAATGC NdeI
lmbB1-Duet-rev	TATCTCGAGTTATCGGGGGCCGTCCGGAACGTAG XhoI
ant6-for	TATGAATTCAATGACGCCGGCGGAGCGACCGC EcoRI
ant6-rev	TATAAGCTTTTACTCGCATACCGCGTACGCGTGC HindIII
ant12-for	TATCATATGAACACGCCGAGCACACCCGC NdeI
ant12-rev	TATGGTACCTTACGTTCCCGCTCCTGGAAAGTAG KpnI
sibY-for	TATGAATTCAATGCTGTCGCGTCCCGAACTGC EcoRI
sibY-rev	TATAAGCTTTTAGCGGACGATCCCGTACGCCTGC HindIII
sibV-for	TATCATATGCCGCTCCGCGCCCATCACG NdeI
sibV-rev	TATGGTACCTTATCGGGGTCCGTCCGGCACGTG KpnI
lmbA2991-for	TATGAATTCAATGTTCACCACCCGACCCACTCTC EcoRI
lmbA2991-rev	TATAAGCTTTTAGCGTCCTACCGCGTACCCCTGC HindIII
lmbA2991-large-for	TATGAATTCAATGTTCACCACCCGACCCACTCTC EcoRI
lmbA2991-large-rev	TATAAGCTTTTAGTCGCCCCGGGTGGAGCCGTCG HindIII
lmbA2991-small-for	TATCATATGACCTGCCACCTCGACGTCGTCG NdeI
lmbA2991-small-rev	TATCTCGAGTTAGCGTCCTACCGCGTACCCCTGC XhoI
ant6-D429A-for	ACGGCGGCGAAGGGCGCAACCTGCACGGTCACCGCC
ant6-D429A-rev	GGCGGTGACCGTGCAGGTTGCGCCCTTCGCCGCCGT
ant6-T430A-for	ACGGCGGCGAAGGGCGACGCATGCACGGTCACCGCC
ant6-T430A-rev	GGCGGTGACCGTGCATGCGTCGCCCTTCGCCGCCGT
ant6-D429A& T430A-for	ACGGCGGCGAAGGGCGCAGCATGCACGGTCACCGCC

ant6-D429A& T430A-rev	GGCGGTGACCGTGCATGCTGCGCCCTTCGCCGCCGT
ant6-T430C-for	ACGGCGGCGAAGGGCGACTGCTGCACGGTCACCGCC
ant6-T430C-rev	GGCGGTGACCGTGCAGCAGTCGCCCTTCGCCGCCGT
ant6-T430S-for	ACGGCGGCGAAGGGCGACTCATGCACGGTCACCGCC
ant6-T430S-rev	GGCGGTGACCGTGCATGAGTCGCCCTTCGCCGCCGT
lmbA2991-D420A-for	GACGTCGAGGTGGCAGGTTGCGCCCCGGGTGGAGCC
lmbA2991-D420A-rev	GGCTCCACCCGGGGCGCAACCTGCCACCTCGACGTC
lmbA2991-T421A-for	GACGTCGAGGTGGCATGCGTCGCCCCGGGTGGAGCC
lmbA2991-T421A-rev	GGCTCCACCCGGGGCGACGCATGCCACCTCGACGTC
lmbA2991-D420A& T421A-for	GACGTCGAGGTGGCATGCTGCGCCCCGGGTGGAGCC
lmbA2991-D420A& T421A-rev	GGCTCCACCCGGGGCGCAGCATGCCACCTCGACGTC
lmbA2991-T421C-for	GACGTCGAGGTGGCAGCAGTCGCCCCGGGTGGAGCC
lmbA2991-T421C-rev	GGCTCCACCCGGGGCGACTGCTGCCACCTCGACGTC
lmbA2991-T421S-for	GACGTCGAGGTGGCATGAGTCGCCCCGGGTGGAGCC
lmbA2991-T421S-rev	GGCTCCACCCGGGGCGACTCATGCCACCTCGACGTC
lmbA2991 HD L-D420A&S-for	TGCGGCCGCAAGCTTTTATGCGCCCCGGGTGGAGCC
lmbA2991 HD L-D420A&S-rev	GGCTCCACCCGGGGCGCATAAAAGCTTGCGGCCGCA
lmbA2991 HD L&S-T1A-for	GACGTCGAGGTGGCATGCCATATGTATATCTCCTTC
lmbA2991 HD L&S-T1A-rev	GAAGGAGATATACATATGGCATGCCACCTCGACGTC
lmbA2991 HD L&S-T1C-for	GACGTCGAGGTGGCAGCACATATGTATATCTCCTTC
lmbA2991 HD L&S-T1C-rev	GAAGGAGATATACATATGTGCTGCCACCTCGACGTC
lmbA2991 HD L&S-T1S-for	GACGTCGAGGTGGCATGACATATGTATATCTCCTTC
lmbA2991 HD L&S-T1S-rev	GAAGGAGATATACATATGTCATGCCACCTCGACGTC

Supplementary Methods

Materials and methods

Materials, bacterial strains and plasmids. Biochemicals and media were purchased from Sinopharm Chemical Reagent Co. Ltd. (China) or Oxoid Ltd. (UK) unless stated. Enzymes were purchased from Takara Biotechnology Co. Ltd. (China) except Taq DNA polymerase from Dingguo Co. Ltd. (China). Chemical compounds and reagents were purchased from Sigma-Aldrich Co. (USA), TCI Development Co. Ltd. (Japan) and J&K Scientific Ltd. (China). Tetrahydrofuran (THF) and toluene were distilled from sodium prior to use. Dichloromethane and pyridine were distilled from calcium hydride. All reactions involving moisture sensitive reactants were executed under argon atmosphere using oven dried and/or flame dried glassware. All other solvents, reagents and chemicals were used as purchased unless stated otherwise. Bacteria strains and plasmids used in this study are listed in Supplementary Table 1. Primers used in this study are summarized in Supplementary Table 2.

Analysis. High performance liquid chromatography (HPLC) analysis was carried out on Agilent 1200 HPLC system (Agilent Technologies Inc., USA). Electrospray ionization mass spectrometry (ESI-MS) was performed on a Thermo Fisher LTQ Fleet ESI-MS spectrometer (Thermo Fisher Scientific Inc., USA), and the data were analyzed using Thermo Xcalibur software. ESI-high resolution MS (ESI-HR-MS) analysis was carried out on 6230B Accurate-Mass TOF LC/MS System or 6530 Accurate-Mass Q-TOF LC/MS System (Agilent Technologies Inc., USA) and the

data were analyzed using Agilent MassHunter Qualitative Analysis software. Electron impact mass spectrometry (EI-MS) was performed on Agilent Technologies 5793N. EI-HR-MS was carried out on Waters Micromass GCT Premier. HR-MS analysis of LmbA2991 was performed on Triple-TOF 5600$^+$ (AB Sciex, USA). *N*-terminally sequencing of LmbA2991 was conducted on PPSQ-33A Protein Sequencer System (Shimadzu, Japan). NMR data were recorded on the Bruker AV500 spectrometers (Bruker Co. Ltd, Germany) or on the Agilent 500 MHz Premium Compact+ NMR spectrometer (Agilent Technologies Inc., USA).

Gene inactivation and complementation

In-frame deletion of *lmbA*. The primers lmbA-L-for and lmbA-L-rev were used to amplify a 1.8 kb DNA fragment from *S. lincolnensis*, which was cloned into pMD19-T to yield pLL2001. On the other hand, the primers lmbA-R-for and lmbA-R-rev were used to obtain a 1.7 kb fragment, insertion of which into pMD19-T led to the generation of pLL2002. The 1.8 kb *Eco*RI-*Xba*I fragment from pLL2001 and 1.7 kb *Xba*I-*Hin*dIII fragment from pLL2002 were recovered and then cloned into the *Eco*RI-*Hin*dIII site of pKC1139, giving the recombinant plasmid pLL2003, in which 1185 bp in-frame coding region of *lmbA* was deleted. To transfer pLL2003 into the lincomycin producing strain *S. lincolnensis*, conjugation between *E. coli* ET12567 (pUZ8002) -*Streptomyces* was carried out. The colonies that were apramycin-resistant at 37 °C were identified as the integrating mutants, in which a single-crosssover homologous recombination event took place. These mutants were further cultivated on the MS agar plate for five rounds in the absence of apramycin. The genotypes of resulting strains that were apramycin-sensitive were confirmed by

PCR amplification (with the primers lmbA-for and lmbA-rev, Supplementary Fig. 1a), leading to identification of the recombinant strain LL2001 for *lmbA* inactivation.

In-frame deletion of *lmbA* and *lmbA2991*. The primers lmbA2991-L-for and lmbA2991-L-rev were used to amplify a 2.2 kb DNA fragment from *S. lincolnensis*, which was cloned into pMD19-T to yield pLL2004. On the other hand, the primers lmbA2991-R-for and lmbA2991-R-rev were used to obtain a 2.0 kb fragment, insertion of which into pMD19-T led to the generation of pLL2005. The 2.2 kb *Eco*RI-*Xba*I fragment from pLL2004 and 2.0 kb *Xba*I-*Hin*dIII fragment from pLL2005 were recovered and then cloned into the *Eco*RI-*Hin*dIII site of pKC1139, giving the recombinant plasmid pLL2006, in which 1254 bp in-frame coding region of *lmbA2991* was deleted. pLL2006 was transferred into LL2001 (Δ*lmbA* mutant). Following the procedure described above for double crossover mutant identification, we constructed the recombinant strain LL2002 for *lmbA* and *lmbA2991* double inactivation. The genotype of LL2002 was confirmed by PCR amplification by using the primers lmbA-for, lmbA-rev and lmbA2991-for, lmbA2991-rev (Supplementary Fig. 1b).

Homologous complementation of the Δ*lmbA* mutant *in trans*. A *lmbA* containing fragment was amplified by PCR using the primers lmbA-com-for, lmbA-com-rev and then cloned into pMD19-T to yield pLL2007. After sequencing, the *BamH*I/*Xba*I 2136 bp *lmbA* fragment was recovered from pLL2007 and ligated to a 0.45-kb *EcoR*I/*BamH*I fragment from pWHM79, and the resulting product was ligated into the *EcoR*I/*Xba*I site of pKC1139, yielding the recombinant plasmid pLL2008, in which *lmbA* was under the control of the constitutive promoter *PermE**. pLL2008

was introduced into LL2001 (Δ*lmbA* mutant) by conjugation, generating the corresponding recombinant strain LL2003 that expressed *lmbA in trans*.

Heterogenous complementation of the Δ*lmbA* mutant *in trans*. An *ant6* containing fragment was amplified by PCR using the primers ant6-com-for, ant6-com-rev and then cloned into pMD19-T to yield pLL2009. After sequencing, the *Bgl*II/*Xba*I 2196 bp *ant6* fragment was recovered from pLL2009 and ligated to a 0.45-kb *EcoR*I/*BamH*I fragment from pWHM79, and the resulting product was ligated into the *EcoR*I/*Xba*I site of pKC1139, yielding the recombinant plasmid pLL2010, in which *ant6* was under the control of the constitutive promoter *PermE**. pLL2010 was introduced into LL2001 (Δ*lmbA* mutant) by conjugation, generating the corresponding recombinant strain LL2004 that expressed *ant6 in trans*.

Fermentation and analysis

Fermentation. *S. lincolnensis* wild-type strain or its derivatives was spread on agar plates, which was composed of 19 g of starch, 5 g of soybean meal, 0.5 g of K_2HPO_4, 0.5 g of $MgSO_4·7H_2O$, 1.0 g of KNO_3, 0.5 g of NaCl, 0.01 g of $FeSO_4·7H_2O$ and 20.0 g of agar per litre (pH 7.0~7.5), and then incubated at 30 °C for sporulation and growth. Approximately 1 cm^2 of the sporulated agar of *S. lincolnensis* was cut, chopped, and inoculated into 25 mL of the seed medium, which was composed of 20 g of starch, 10 g of glucose, 10 g soybean of meal, 30 g of corn steep liquor, 1.5 g of $(NH_4)_2SO_4$ and 5 g of $CaCO_3$ per litre (pH 7.0~7.5). After incubation at 28 °C and 220 rpm for 36 h, 5 mL of the seed culture broth was transferred into 50 mL of the fermentation

medium, which was composed of 100 g of glucose, 25 g of soybean meal, 2 g of corn steep liquor, 8 g of $(NH_4)_2SO_4$, 0.2 g of KH_2PO_4, 8 g of $NaNO_3$, 5 g of NaCl and 8 g of $CaCO_3$ per litre (pH 7.0~7.5). Further incubation was carried out at 28 °C and 220 rpm for 7 days.

Analysis. For product examination, 0.5 mL of the supernatant of each fermentation broth was mixed with an equal volume of methanol, and after centrifugation to remove the residue, the supernatant was subjected to HPLC-ESI-MS analysis on a phenomenex column (Luna 5μ C18(2) 100A, 4.60×250 mm, 5 micron, phenomenex Inc., USA) by gradient elution of solvent A (H_2O containing 10 mM NH_4OAc) and solvent B (CH_3CN) at a flow rate of 1 mL/min over a 25-min period as follows: t=0 min, 10% B; t=9 min, 10% B; t=20 min, 60% B; t=25 min, 60% B (mAU at 210 nm).

Construction the plasmids used for L-DOPA transformation in *E. coli*

Plasmid pLL2021 (pACYCDuet-1+*ant6*+*ant12*). A 1866 bp DNA fragment containing *ant6* was amplified by PCR using the primers ant6-for and ant6-rev and then cloned into pMD19-T to yield pLL2019. A 510 bp DNA fragment containing *ant12* was amplified by PCR using the primers ant12-for and ant12-rev and then cloned into pMD19-T to yield pLL2018. After sequencing to validate the fidelity, the *ant6* fragment recovered from pLL2019 was inserted into the *Eco*RI-*Hind*III site of pACYCDuet-1 (Novagen) to yield the recombinant plasmid pLL2020. The *ant12* fragment recovered from pLL2018 was inserted into the *Nde*I-*Kpn*I site of pLL2020 to yield the recombinant plasmid pLL2021.

Plasmid pLL2022 (pACYCDuet-1+*ant12*). The *ant12* fragment recovered from pLL2018 was inserted into the *Nde*I-*Kpn*I site of pACYCDuet-1 to yield the recombinant plasmid pLL2022.

Plasmids pLL2016 (pACYCDuet-1+*lmbA*+*lmbB1*), pLL2017 (pACYCDuet-1+*lmbB1*), pLL2026 (pACYCDuet-1+*sibY*+*sibV*) and pLL2027 (pACYCDuet-1+*sibV*) were constructed using the same method as for pLL2021 and pLL2022.

Protein expression and purification

LmbB1. A 477 bp DNA fragment containing *lmbB1* was amplified by PCR using the primers lmbB1-28a-for and lmbB1-28a-rev and then cloned into pMD19-T to yield pLL2011. After sequencing to validate the fidelity, the *lmbB1* fragment recovered from pLL2011 was inserted into the *Nde*I-*Hind*III site of pET28a(+) to yield the recombinant plasmid pLL2012. The resulting plasmid pLL2012 was transferred into *E. coli* BL21 (DE3) for expression. LmbB1 was expressed, purified and reconstituted according to a previously described method[1,7].

Ant6. Plasmid pLL2021 was transferred into *E. coli* BL21 (DE3) for expression. Ant6, fused to an *N*-terminal 6 x His tag was expressed at 16 °C for 40 h with 100-μM isopropyl-β-D-thiogalactopyranoside (IPTG, added at OD_{600}=0.6) induction and shaking at 220 rpm. Cells were harvested by centrifugation at 4 °C and re-suspended in lysis buffer containing 50-mM K_2HPO_4 (pH 8.0), 300-mM NaCl, 5-mM imidazole and 10% (v/v) glycerol. After

disruption by a low-temperature ultra-high-pressure cell disrupter, the insoluble material was removed by centrifugation at 15000 g at 4 °C. The soluble fraction was subjected to purification using a HisTrap FF column (GE Healthcare, USA) according to the manufacturer's protocol. The elution fraction containing the recombinant protein was desalted using a PD-10 Desalting Column (GE Healthcare, USA) into storage buffer (50-mM K_2HPO_4 (pH 8.0), 100-mM NaCl, 10% (v/v) glycerol and 1-mM DTT). The resulting protein was concentrated and stored at -80 °C. The purity of the protein was determined by 10% sodium dodecyl sulfate polyacrylamide gel electrophoresis (SDS–PAGE) analysis, and the concentration was determined by the Bradford assay using bovine serum albumin (BSA) as the standard.

LmbA2991. A 1854 bp DNA fragment containing *lmbA2991* was amplified by PCR using the primers lmbA2991-for and lmbA2991-rev and then cloned into pMD19-T to yield pLL2028. A 477 bp DNA fragment containing *lmbB1* was amplified by PCR using the primers lmbB1-Duet-for and lmbB1-Duet-rev and then cloned into pMD19-T to yield pLL2013. After sequencing to validate the fidelity, the *lmbA2991* fragment recovered from pLL2028 was inserted into the *EcoR*I-*Hind*III site of pACYCDuet-1 to yield the recombinant plasmid pLL2029. The *lmbB1* fragment recovered from pLL2013 was inserted into the *Nde*I-*Xho*I site of pLL2029 to yield the recombinant plasmid pLL2030. The resulting plasmid pLL2030 was transferred into *E. coli* BL21 (DE3) for expression. LmbA2991 fused to an *N*-terminal 6 x His tag was expressed and purified following a procedure similar to that used for Ant6.

LmbA2991 HD L&S. (co-expression of the large and small subunits). A 1260 bp DNA

fragment containing the *lmbA2991* large subunit was amplified by PCR using the primers lmbA2991-for and lmbA2991-large-rev and then cloned into pMD19-T to yield pLL2031. A 594 bp DNA fragment containing the *lmbA2991* small subunit was amplified by PCR using the primers lmbA2991-small-for and lmbA2991-rev and then cloned into pMD19-T to yield pLL2032. After sequencing to validate the fidelity, the *lmbA2991* large subunit fragment recovered from pLL2031 was inserted into the *Eco*RI-*Hind*III site of pACYCDuet-1 to yield the recombinant plasmid pLL2033. The *lmbA2991* small subunit fragment recovered from pLL2032 was inserted into the *Nde*I-*Xho*I site of pLL2033 to yield the recombinant plasmid pLL2034. The resulting plasmid pLL2034 was transferred into *E. coli* BL21 (DE3) for expression. The heterodimer fused to an *N*-terminal 6 x His tag of the large subunit was expressed and purified following a procedure similar to that used for Ant6.

Compound isolation and derivatization

Isolation and purification of compound 5 from the LmbB1 and Ant6-catalysed reaction. The assay was scaled up and carried out at 30 °C for 6 h in 15 mL of mixture containing 100 mM K_2HPO_4 buffer (pH 8.0), 2 mM L-DOPA, 10 mM LmbB1 and 10 mM Ant6. The assays were quenched by adding an equal volume of methanol. After centrifugation (5 min at 13000 g) to remove proteins, the supernatant was concentrated in vacuo and then loaded onto a reversed phase silica gel column (RP-18, 25-40 μm, Merck, USA) by eluting of 95% H_2O (containing 20 mM NH_4OAc) and 5% CH_3OH mixture. According to HPLC analysis, the fractions containing the target compound were combined, evaporated in vacuo and repeated the flash chromatography for

once. Because of the imine group, this compound is not very stable and should be purified quickly.

Derivatization of LmbB1 and Ant6 (LmbA2991) biochemical assay. To 0.6 mL of assay reaction in 1.5 mL Eppendorf tube, hydrochloric acid was added to adjust pH<1.0. The tube was vortex-mixed and the contents extracted four times with 0.6 mL of ethyl acetate by vortex-mixing for exactly 2 min. The phases were separated by centrifugation (5 min at 13000 g) and the four ethyl acetate extracts pooled and dried over anhydrous sodium carbonate. The ethyl acetate was evaporated in vacuo to a crude extract. 50 μL methoxyamine hydrochloride (40 mg/mL in anhydrous pyridine) was added to the tube, mixed and reacted in 30 °C for 1 h. Then 50 μL anhydrous pyridine and 50 μL *N*-(*tert*-Butyldimethylsilyl)-*N*-methyltrifluoroacetamide (MTBSTFA) were added to the tube and reacted in 70 °C for 30 min. After centrifugation (5 min at 13000 g), the supernatant was analyzed by GC-EI-MS and GC-EI-HR-MS.

Chemical synthesis

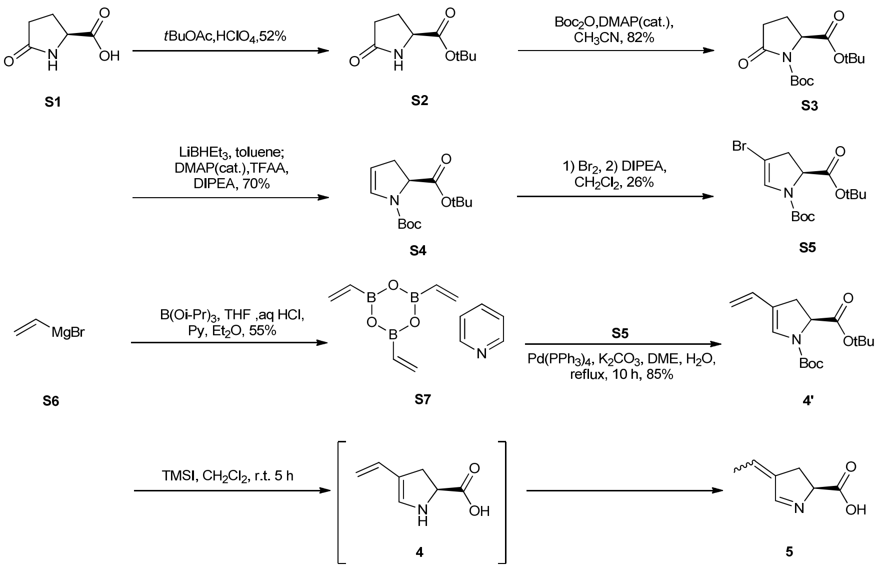

Compound **S5** and **S7** were synthesized according to the methods previously described[8-10]. **4'** and **5** were synthesized as given below.

(*S*)-*tert*-butyl 5-oxopyrrolidine-2-carboxylate (S2). White solid, **^{1}H-NMR** (500 MHz, $CDCl_3$): δ/ppm=1.47 (s, 9H), 2.13-2.18 (m, 1H), 2.32-2.41 (m, 3H), 4.11-4.13 (m, 1H), 6.29 (s_{br}, 1H). **^{13}C-NMR** (125 MHz, $CDCl_3$): δ/ppm=24.97 (-), 28.09 (+), 29.49 (-), 56.19 (+), 82.51 (C_{quart}), 171.19 (C_{quart}), 177.96 (C_{quart}). ESI-HR-MS $(C_9H_{16}NO_3)^+$: calcd. 186.1125, found 186.1128.

(*S*)-di-*tert*-butyl 5-oxopyrrolidine-1,2-dicarboxylate (S3). Yellow solid. **^{1}H-NMR** (500 MHz, $CDCl_3$): δ/ppm=1.47 (s, 9H), 1.49 (s, 9H), 1.97 (ddt, *J*=13.5, *J*=10.0, *J*=2.5 Hz, 1H), 2.22-2.31 (m, 1H), 2.41-2.47 (m, 1H), 2.59 (dt, *J*=17.5, *J*=10 Hz, 1H), 4.46 (dd, *J*=8.5, *J*=2.5 Hz, 1H). **^{13}C-NMR** (125 MHz, $CDCl_3$): δ/ppm=21.77 (-), 27.97 (+), 28.03 (+), 31.25 (-), 59.72 (+), 82.38 (C_{quart}), 83.42 (C_{quart}), 149.43 (C_{quart}), 170.48 (C_{quart}), 173.65 (C_{quart}). EI-HR-MS ([M^+-CH_3], $C_{13}H_{20}NO_5$): calcd. 270.1341, found 270.1339.

(*S*)-di-*tert*-butyl 2,3-dihydro-1*H*-pyrrole-1,2-dicarboxylate (S4). Faint yellow oil. **^{1}H-NMR** (500 MHz, acetone-d_6): δ/ppm=1.43, 1.44, 1.47, 1.48 (4×s, 18H), 2.53-2.62 (m, 1H), 2.99-3.12 (m, 1H), 4.43-4.48 (m, 1H), 4.91-4.95 (m, 1H), 6.50-6.56 (m, 1H). **^{13}C-NMR** (125 MHz, acetone-d_6); δ/ppm=27.15 (+), 27.39 (+), 36.26 (-), 59.59 (+), 80.75 (C_{quart}), 81.67 (C_{quart}), 105.72 (+), 130.91 (+), 152.18 (C_{quart}), 171.60 (C_{quart}). EI-HR-MS ($C_{14}H_{23}NO_4$): calcd. 269.1627, found 269.1624.

***(S)*-di-*tert*-butyl 4-bromo-2,3-dihydro-1*H*-pyrrole-1,2-dicarboxylate (S5).** Yellow oil. **^{1}H-NMR (**500 MHz, $CDCl_3$): δ/ppm=1.44, 1.47 (2×s, 18H), 2.75-2.79 (m, 1H), 3.19-3.31 (m, 1H), 4.49 (dd, *J*=12.0, *J*=4.5 Hz, 0.5H), 4.56 (dd, *J*=12.0, *J*=4.5 Hz, 0.5H), 6.61 (s, 0.5H), 6.74 (s, 0.5H). **^{13}C-NMR** (125 MHz, $CDCl_3$), δ/ppm=28.06 (+), 28.09 (+), 28.31 (+), 28.38 (+), 40.50 (-), 41.66 (-), 59.26 (+), 59.59 (+), 81.50 (C_{quart}), 82.10 (C_{quart}), 97.10 (C_{quart}), 130.03 (+), 150.64 (C_{quart}), 169.90 (C_{quart}). EI-HR-MS ($C_{14}H_{22}NO_4{}^{79}Br$): calcd. 347.0732, found 347.0735.

2,4,6-trivinyl-1,3,5,2,4,6-trioxatriborinane-pyridine complex (S7). Yellow solid. **^{1}H-NMR** (500 MHz, $CDCl_3$): δ/ppm=5.85 (dd, *J*=8.0, *J*=4.5 Hz, 4H), 5.96-6.09 (m, 8H), 7.64 (t, *J*=7.0 Hz, 2H), 8.06 (t, *J*=8.0 Hz, 1H), 8.85 (d, *J*=5.5 Hz, 2H). **^{13}C-NMR** (125 MHz, $CDCl_3$): δ/ppm=125.72, 132.04, 141.15, 143.88. EI-HR-MS ($C_6H_9O_3{}^{10}B_3$): calcd. 159.0940, found 159.0943.

(*S*)-di-*tert*-butyl 4-vinyl-2,3-dihydro-1*H*-pyrrole-1,2-dicarboxylate (4'). Compound **S5** (1.716 g, 4.928 mmol) was dissolved in DME (40 mL), and $Pd(PPh_3)_4$ (0.570 g, 0.493 mmol, 10% eq.) was added. The mixture was stirred at r.t. under N_2 for 20 min. Anhydrous K_2CO_3 (0.745 g, 5.421 mmol, 1.1 eq.), H_2O (9.2 mL), and **S7** (1.406 g, 5.842 mmol, 1.18 eq.) were added, and the mixture was heated under reflux under N_2 for 10 h. The mixture was cooled to r.t. and extracted with Et_2O (3 × 30 mL). The combined organic layers were dried (Na_2SO_4) and concentrated. The crude material was purified by flash chromatography over silica gel (Hex–EtOAc, 40:1) to give the title product **4'** as a yellow oil. **^{1}H-NMR** (500 MHz, $CDCl_3$): δ/ppm=1.45, 1.46, 1.47 (3×s, 18H), 2.65 (td, *J*=12.5, *J*=4.5 Hz, 1H), 3.03-3.15 (m, 1H), 4.50 (dd, *J*=12, *J*=5 Hz, 0.5H), 4.56 (dd, *J*=12, *J*=5 Hz, 0.5H), 4.84 (t, *J*=17 Hz, 1H), 4.94 (t, *J*=10.5 Hz, 1H), 6.40-6.49 (m, 1H), 6.53 (s,

0.5H), 6.68 (s, 0.5H). **^{13}C-NMR** (125 MHz, $CDCl_3$): δ/ppm=28.05 (+), 28.11 (+), 28.32 (+), 28.42 (+), 34.31 (-), 59.15 (+), 59.41 (+), 81.13 (C_{quart}), 81.63 (C_{quart}), 111.44 (-), 111.72 (-), 119.39 (+), 119.61 (+), 128.64 (C_{quart}), 128.83 (C_{quart}), 130.26 (+), 130.36 (+), 151.34 (C_{quart}), 170.68 (C_{quart}). EI-HR-MS ($C_{16}H_{25}NO_4$): calcd. 295.1784, found 295.1778.

(*S*)-4-vinyl-2,3-dihydro-1*H*-pyrrole-2-carboxylic acid (5). Compound **4'** (1.5010 g, 5.0851 mmol) was dissolved in anhydrous CH_2Cl_2 (30 mL), and stirred in ice water. TMSI (2.89 mL, 20.3404 mmol, 4.0 eq.) was added slowly to the solution under N_2. After stirred for 30 min, the ice water was removed and stirred for another 5 h. Then the reaction system was poured slowly into saturated $NaHCO_3$ aqueous solution to remove unreacted TMSI and HI. The organic phase was separated and extracted with H_2O (3 x 30 mL). The aqueous phase was combined and concentrated in vacuo and then loaded onto a reversed phase silica gel column (RP-18, 25-40 μm, Merck, USA) by eluting of 95% H_2O (containing 20 mM NH_4OAc) and 5% CH_3OH mixture. According to HPLC analysis, the fractions containing the target compound were combined, evaporated in vacuo and repeated the flash chromatography for twice until the pure compound was obtained as a redbrown oil. Because of the imine group, this compound is not very stable and should be synthesized and purified quickly. **^{1}H-NMR** (500 MHz, D_2O): δ/ppm=2.00 (dd, *J*=6, *J*=2 Hz, 3H), 2.92 (dt, *J*=17, *J*=2 Hz, 1H), 3.24-3.29 (m, 1H), 5.00 (dd, *J*=10, *J*=4.5 Hz, 1H), 7.07-7.11 (m, 1H), 8.62 (s, 1H). **^{13}C-NMR** (125 MHz, D_2O): δ/ppm=13.21 (+), 24.97 (-), 68.78 (+), 135.12 (+), 144.81 (C_{quart}), 168.01 (+), 171.79 (C_{quart}). ESI-HR-MS $[C_7H_{10}NO_2]^+$: calcd. 140.0706, found 140.0705.

Protein sequence of LmbA2991

LmbA2991 which is outside of lincomycin biosynthetic gene cluster contains 617 amino acids, with 420 amino acids for large subunit in the *N* terminal and 197 amino acids for small subunit in the *C* terminal. The demarcation point of large and small subunit is shown in red.

MFTTRPTLQGTFGMVSSTHWLASQSAMAVLEDGGNAYDAAVAGAFVLHVVEPHLNGPA
GEVPILLAPAGGEVRVLCGQGVAPAGATVAHYKGLGLDLVPGTGPLAAAVPGAFDAWML
LLRDHGTKPLADVLKYAVGYAEHGHAPVENVGVTVETVRELFETEWTTSADVYLPGGKA
PRPGELLRNPTLAATWKRLLAEVAGAGDREAQIEAAREVWRTGFIAEALVRQARRPTMDT
SGERHTGTLTAADLAGWSATYEAPATYDWNGWTVCKAGPWSQGPVLLQQLALLPPELPE
YGSADYVHLLVEGCKLAMADREAWYGDAAEVPLDELLSAEYNAGRRELVGDKASHELR
PGSPGGRTARLSAHADLVATGEPGFDPLGAGEPTAAMGAGEPTVAKLPASPVPGEPDVAAD
GSTRG**DT**CHLDVVDRWGNMVAATPSGGWLQSNPVVPELGFPLGTRLQMTWLEEGLPNS
LTPGRRPRTTLTPSIALRDGIPVMAFGTPGGDQQDQWQLHFFLAVALRARVRGGLDLQGAI
DAPNWHNDSFPGSFYPRGMRPGSVTVEARMDPGIAAELRRRGHEVTVGPPWSEGRLCAV
ARDPRTGILSAAANPRGMQGYAVGR

Supplementary References

1. Colabroy, K. L. *et al.* Biochemical characterization of L-DOPA 2,3-dioxygenase, a single-domain type I extradiol dioxygenase from lincomycin biosynthesis. *Arch. Biochem. Biophys.* **479**, 131-138 (2008).
2. Saha, S., Li, W., Gerratana, B. & Rokita, S. E. Identification of the dioxygenase-generated intermediate formed during biosynthesis of the dihydropyrrole moiety common to anthramycin and sibiromycin. *Bioorg. Med. Chem.* **23**, 449-454 (2015).
3. Colabroy, K. L. Tearing down to build up: Metalloenzymes in the biosynthesis lincomycin, hormaomycin and the pyrrolo [1,4]benzodiazepines. *BBA-proteins proteom.* **1864**, 724-737 (2016).
4. MacNeil, D. J. *et al.* Analysis of *Streptomyces avermitilis* genes required for avermectin biosynthesis utilizing a novel integration vector. *Gene* **111**, 61-68 (1992).
5. Keiser, T., Bibb, M. J., Buttner, M. J., Chater, K. F. & Hopwood, D. A. *Practical Streptomyces Genetics* (John Innes Foundation, 2000).
6. Shen, B. & Hutchinson, C. R. Deciphering the mechanism for the assembly of aromatic polyketides by a bacterial polyketide synthase. *Proc. Natl. Acad. Sci. USA* **93**, 6600-6604 (1996).
7. Colabroy, K. L., Smith, I. R., Vlahos, A. H., Markham, A. J. & Jakubik, M. E. Defining a kinetic mechanism for L-DOPA 2,3 dioxygenase, a single-domain type I extradiol dioxygenase from *Streptomyces lincolnensis*. *BBA-proteins proteom.* **1844**, 607-614 (2014).
8. Gross, U., Nieger, M. & Bräse, S. Stereoselective synthesis of the epicoccin core. *Org. Lett.*

11, 4740-4742 (2009).

9. Izgu, E. C. & Hoye, T. R. *o*-(Trialkylstannyl)anilines and their utility in Migita–Kosugi–Stille cross-coupling: direct introduction of the 2-aminophenyl substituent. *Tetrahedron Lett.* **53**, 4938-4941 (2012).

10. Duan, X.-F., Shen, G. & Zhang, Z.-B. A concise synthesis of natural benzofuran neolignans and analogues. *Synthesis* **15**, 2547-2552 (2010).
